# Enzymatic oxygen reduction dominates overpotential-driven thermogenesis in mitochondria

**DOI:** 10.1039/d5sc06693j

**Published:** 2026-03-30

**Authors:** Nuning Anugrah Putri Namari, Mo Yan, Junji Nakamura, Kotaro Takeyasu

**Affiliations:** a International Institute for Carbon-Neutral Energy Research (I^2^CNER), Kyushu University 744 Motooka, Nishi-ku, Fukuoka-shi Fukuoka 819-0395 Japan nakamura.junji.700@m.kyushu-u.ac.jp; b Department of Materials Science, Institute of Pure and Applied Sciences, University of Tsukuba 1-1-1 Tennodai Tsukuba Ibaraki 305-8573 Japan; c Tsukuba Research Center for Energy and Materials Science, University of Tsukuba 1-1-1 Tennodai Tsukuba Ibaraki 305-8573 Japan; d Graduate School of Science and Technology, University of Tsukuba 1-1-1 Tennodai Tsukuba Ibaraki 305-8573 Japan; e Institute for Catalysis, Hokkaido University N21W10 Sapporo Hokkaido 001-0021 Japan takeyasu@cat.hokudai.ac.jp

## Abstract

Understanding how chemical energy dissipates as heat in non-equilibrium redox systems is a fundamental problem in physical chemistry. While this phenomenon is well described in electrochemical systems such as fuel cells, its role in biological enzymatic systems remains underexplored. Mitochondrial thermogenesis has long been attributed to proton leakage, which correlates with heat generation but lacks a clearly defined physical mechanism. In fact, catalytic reactions—whether occurring on inorganic electrodes or in biological enzymes—inevitably require finite overpotentials, and quantifying these losses demands a site-specific kinetic descriptor. To this end, we introduce the electron transfer frequency (ETF), directly analogous to the turnover frequency (TOF) in heterogeneous catalysis, as a means to analyze enzymatic electron-transfer processes at the single-site level. Using ETF as the central descriptor, we develop a chemistry-based framework that models intracellular heat production as the dissipation of Gibbs free energy through enzymatic overpotentials in the mitochondrial electron transport chain, analogous to Joule heat in fuel cells. By treating each respiratory complex as a resistive kinetic step and calibrating the model with experimentally measured electrochemical parameters, we estimate that 45–71% of respiration energy is dissipated as heat. Among these, complex IV alone contributes over 70% of the total dissipation, establishing it as the primary thermogenic site. This framework reproduces reported heat-to-respiration ratios across diverse cell types and demonstrates that overpotential dissipation, rather than proton leakage, represents a major and quantifiable pathway of heat generation. More broadly, it shows that analytical principles of electrocatalysis can be predictively extended to biological redox systems, establishing a common physical chemistry basis for energy dissipation in both.

## Abstract 2

Understanding how chemical energy dissipates as heat in non-equilibrium redox systems is a fundamental problem in physical chemistry.

## Graphical abstract

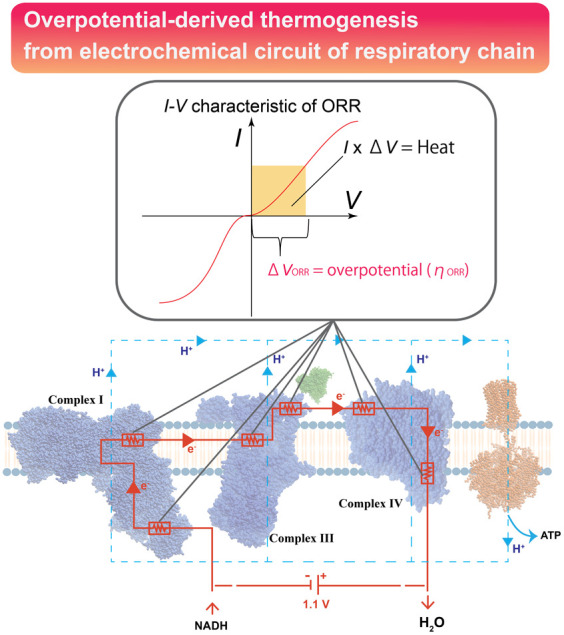

## Introduction

Understanding how energy dissipates in systems where electron transfer reactions proceed under non-equilibrium conditions—ranging from electrochemical reactors to biological enzymatic system—is a central challenge in physical chemistry and chemical physics. In electrochemical systems, energy losses manifest as heat due to overpotentials, that is, excess driving forces beyond the thermodynamic potential required to drive the reactions. While this concept is well established in the context of fuel cells and electrode kinetics, its implications for biological energy transduction, particularly mitochondrial respiration, remain underexplored.

Mitochondria, the primary site of oxidative phosphorylation, have long been interpreted within the framework of chemiosmotic coupling, in which intracellular thermogenesis is attributed to proton leakage across the inner mitochondrial membrane.^1–5^ Recent studies have substantially clarified the molecular pathways mediating proton leak, including the contribution of adenine nucleotide translocase (ANT) to basal proton conductance^1,2,6^ inducible proton transport, and fatty acids acting as mobile proton carriers that facilitate proton cycling,^7,8^ as well as their physiological roles in thermogenesis and reactive oxygen species (ROS) regulation.^7,9,10^ Despite this progress in identifying the carriers and regulatory pathways of proton leak, a fundamental gap remains in our understanding of how the free energy associated with proton transport is quantitatively converted into heat. In particular, proton currents—typically inferred from H^+^/O ratios or oxygen-consumption measurements^11^—ultimately reflect the underlying electron-transfer flux that sustains proton pumping. This raises the possibility that mitochondrial thermogenesis is governed not solely by proton permeability, but by overpotential-driven energy dissipation inherent to electron-transfer reactions. Accordingly, the present study re-examines mitochondrial heat generation from the perspective of electron-transfer kinetics and the associated irreversible energy dissipation.

Notably, Armstrong and Hirst have highlighted those key mitochondrial enzymes, particularly cytochrome *c* oxidase (complex IV), operate under significant overpotentials even under physiological conditions.^12^ As they previously explained, this provides a mechanistic origin for heat generation in electron-transport enzymes. Using non-concerted proton transfer as an example, an overpotential is required to drive electron transfer to an unstable intermediate, which is then spontaneously protonated, releasing energy as heat. However, this interpretation remains qualitative and does not resolve the quantitative importance of this mechanism within the entire respiratory chain under steady-state conditions, which should be addressed by non-equilibrium thermodynamics. In the present study, we address this limitation by introducing the electron transfer frequency (ETF) as a kinetic descriptor, enabling a quantitative evaluation of overpotential and heat dissipation at the system level. This observation raises a critical thermodynamic implication: if substantial overpotentials exist at enzymatic redox sites, then a portion of the Gibbs free energy is not conserved into the work for proton pumping or ATP synthesis but is instead dissipated as heat. This insight motivates a complementary concept: that mitochondrial thermogenesis arises, at least in part, from irreversible energy losses associated with enzymatic overpotentials—analogous to Joule heating in electrochemical systems.

**Fig. 1:**
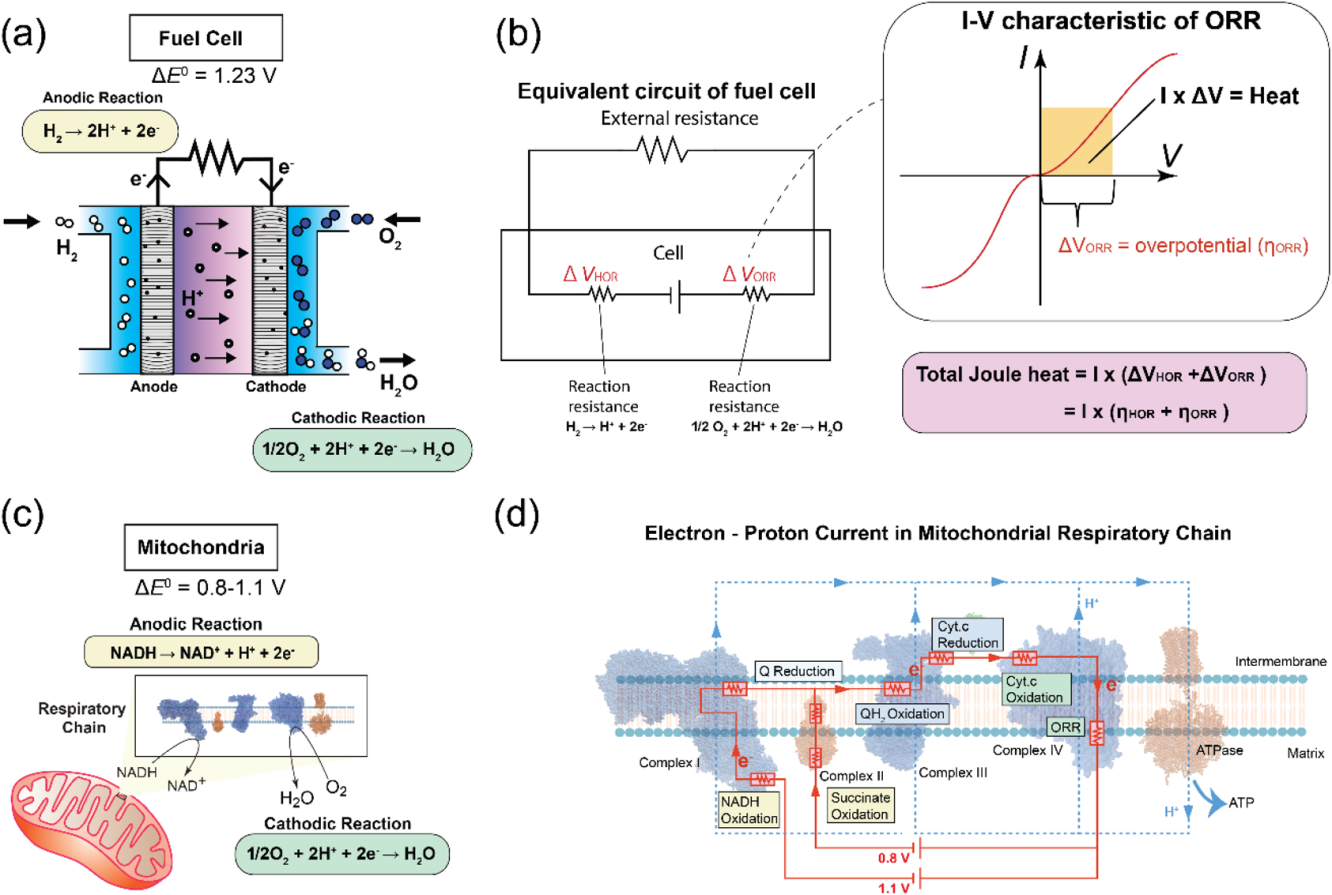
Thermogenesis model based on the electrochemical overpotential of the mitochondrial respiratory chain. (a) Schematic of hydrogen fuel cell, with hydrogen oxidation reaction in the anode and ORR in the cathode similar with mitochondrial electron transport chain. Δ*E*^0^ means redox potential differences between anode and cathode. (b) Equivalent circuit of hydrogen fuel cell and the typical voltage–current curve for the electrochemical reactions. The differences between working potential (*E*) and equilibrium potential (*E*^0^) caused a potential drop (Δ*V*) which corresponds to the overpotential (*η*). The overpotential (*η*) applied to drive the reaction then will be converted to heat (yellow part, *I* × Δ*V*) and the rest potential gain will be used for electricity, the energy partitioning between heat and electricity depends on the current. (c) Mitochondrial Electron Transport Chain (ETC), with NADH oxidation in complex I as the anodic reaction and oxygen reduction reaction (ORR) in complex IV as the cathodic reaction. Δ*E*^0^ means redox potential differences between those two reactions. Δ*E*^0^ = 0.8–1.1 V, corresponding to succinate (CII) and NADH (CI) oxidation; for simplicity only the NADH branch is illustrated. (d) Electron–proton current in the mitochondrial respiratory chain, consisting of complexes I, II, III, and IV and ATPase. Electron current is red, and proton current is blue. Each redox reaction in every complex regarded as a resistor (red color). Here, we assume that resistivity of proton pumping is negligible.

This concept draws a direct analogy to hydrogen fuel cells, in which overpotentials at the anode and cathode are required to overcome kinetic barriers in the hydrogen oxidation reaction (HOR) and the oxygen reduction reaction (ORR), respectively. When these overpotentials (*η*) are multiplied by the current, they generate Joule heat, which represents the irreversible component of energy dissipation. In a similar manner, each enzymatic complex in the mitochondrial electron transport chain (complexes I–IV) can be represented as a resistive element in a non-equilibrium electrochemical circuit. As schematically illustrated in Fig. 1a–d, electrons flow from NADH to molecular oxygen through these complexes, and the kinetic resistances of the enzyme-catalyzed reactions give rise to overpotentials that consume a significant fraction of the available Gibbs free energy (∼1.1 V), which is ultimately dissipated as heat.

The idea that enzymatic redox steps function analogously to electrode reactions is further supported by the increasing recognition that many biological enzymes behave as highly efficient, and in some cases reversible, electrocatalysts. The electrochemical properties of mitochondrial enzymes—including formal potentials, exchange current densities, and kinetic asymmetries—are now sufficiently characterized to allow quantitative analysis based on classical electrode theory. In this context, we introduce the concept of electron transfer frequency (ETF), a frequency-based kinetic descriptor that directly corresponds to the turnover frequency (TOF) widely used in heterogeneous catalysis, defined as the number of product molecules per catalytic active site per second. Unlike conventional bioenergetic metrics, ETF enables site-specific quantification of enzymatic electron-transfer rates, providing a bridge between catalytic kinetics and biological energy dissipation. This approach builds on our previous studies of metal-free carbon electrocatalysts, where TOF analysis clarified the role of pyridinic nitrogen under acidic conditions.^13–15^ Furthermore, our development of mixed-potential-driven models for electrode reactions^16–18^ provides a mechanistic framework analogous to enzymatic redox systems, reinforcing the generality of the present thermogenesis model. In this study, ETF is defined per electron-transfer active site—that is, per individual redox center such as FMN, each Fe–S cluster, the N_2_ center, or the ubiquinone reduction site in complex I. Multi-center complexes therefore contain multiple ETF-defined steps arranged in series and parallel. Crucially, because the respiratory chain operates under continuous steady-state conditions, the net electron flux is conserved along the sequential pathway. Therefore, the steady-state ETF passing through a catalytic active site is equal to that passing through the intermediate electron-transfer relay centers arranged in series. However, we emphasize that this equivalence reflects flux conservation rather than kinetic equivalence: relay centers primarily mediate electron transport, whereas catalytic active sites govern the overall reaction energetics and associated overpotential. ETF applies specifically to electron-transfer transitions, whereas chemical transformation steps should be described using conventional TOF. Combining ETF for redox steps with TOF for chemical steps allows the overall kinetics of multi-step enzymatic cycles to be formulated consistently.

Historically, the electrochemical analogy of the mitochondrial respiratory chain was proposed by Bockris^19,20^ and Berry^21^ in the 1980s and later expanded by Kitasato,^22^ but the limited structural and kinetic data at that time hindered the development of predictive, quantitative models. Recent advances in bioelectrochemical analysis now enable detailed evaluation of enzymatic reaction steps based on experimentally measured kinetic parameters, including exchange current densities, transfer coefficients, and active site concentrations. This work demonstrates that analytical frameworks originally developed for heterogeneous electrocatalysis can be directly extended to biological redox systems, establishing a common physical chemistry basis for energy dissipation in both. In this study, we therefore treat mitochondrial enzymes in analogy to fuel-cell electrodes, while noting that this analogy is necessarily simplified and does not explicitly include dynamic substrate concentrations or protein conformational changes that may influence the results. Nevertheless, sensitivity analyses presented in the SI-Section six to confirm that our main conclusions are robust across a wide range of parameter variations.

On the basis of these advances, we developed an overpotential-based electrochemical framework that enables quantitative estimation of site-specific overpotentials and the corresponding heat dissipation within the mitochondrial respiratory chain. This concept is not framed as a binary alternative to proton leakage-based models but instead offers a complementary framework to quantify the extent of overpotential and its thermogenic consequences under physiologically relevant electron fluxes. If overpotentials are found to be negligible, then the thermogenic contribution from this mechanism would be minimal. However, in any system where overpotentials are non-zero—a thermodynamic inevitability under finite flux—heat production must occur, regardless of whether it is large or small. This approach thus provides a physically grounded framework for quantifying energetic dissipation in biological redox systems, bridging the concepts of electrocatalysis and mitochondrial bioenergetics.

## Result and discussion

### Method to estimate overpotential

To quantify overpotential in the mitochondrial respiratory chain, we employed a multi-step electrochemical analysis based on the Butler–Volmer formalism (eqn (1)).1
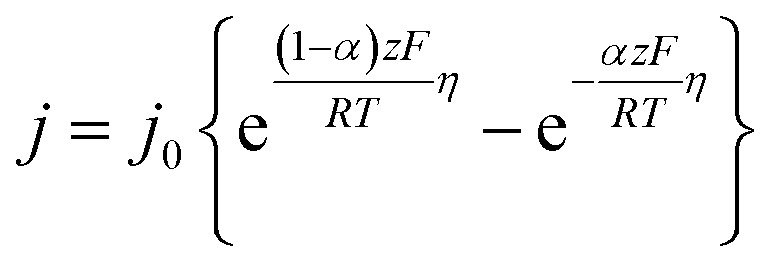


First, we extracted exchange current densities *j*_0_ [A cm^−2^] for each redox step in complexes I–IV by fitting published cyclic voltammograms (CVs)^23–36^ and linear sweep voltammograms (LSVs) data with the Butler–Volmer formalism.^37^ CV was used for (quasi-)reversible reactions, whereas LSV was employed for the electrochemically irreversible ORR at complex IV.

Although the mitochondrial respiratory chain consists of multi-step and multi-electron processes, the Butler–Volmer formalism is widely used as a phenomenological description of potential-dependent interfacial electron-transfer kinetics. It has been successfully applied not only to simple redox reactions but also to enzymatic electrochemical systems involving intramolecular electron transfer, as well as to multi-electron reactions such as HER and ORR.^38–42^

These *j*_0_ values, which reflect intrinsic catalytic activity under equilibrium conditions, were converted to the exchange electron transfer frequency *Γ*_e_0__ [e^−^ per site per s] by normalizing with the surface density of active sites *σ* [mol cm^−2^] according to2
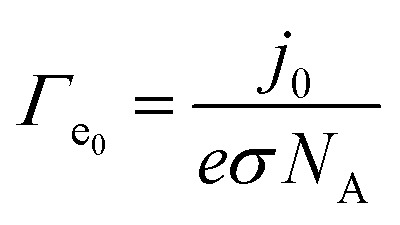
where *e* is the elementary charge and *N*_A_ is Avogadro's number. This frequency-based descriptor *Γ*_e_0__ provides a necessary normalization that converts cellular-level oxygen consumption rates into electron-transfer rates defined per active site. As a result, ETF enables a direct and quantitative analysis of enzymatic electron-transfer kinetics at the single-site level. Next, experimentally measured oxygen consumption rates (OCR), reported in the literature for various cell types (see Table S1), were used to determine steady-state electron transfer frequencies *Γ*_e_ [e^−^ per site per s] by normalizing the OCR values (expressed per unit protein mass) by the number density of complex IV active sites per mg of protein (see SI and Fig. S1).

Finally, using *Γ*_e_ and *Γ*_e_0__ for each complex in the Butler–Volmer eqn (3), we calculated the overpotential *η* [V] for each redox step.3
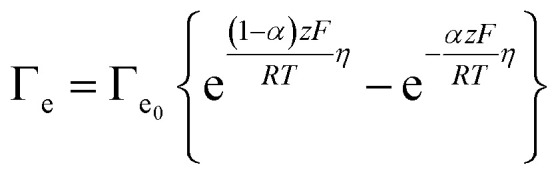


The total energy dissipated as heat in the respiratory chain is given by the sum of the overpotentials multiplied by the ETF, analogous to the total Joule loss in a series electrical circuit.

**Fig. 2:**
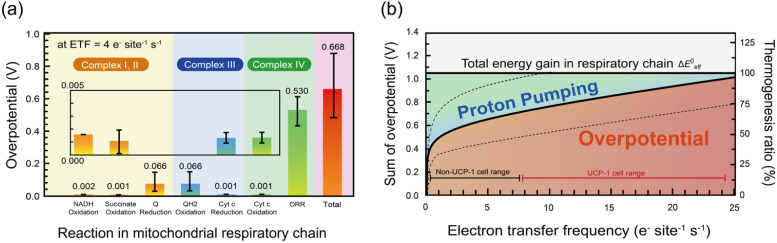
Estimated overpotentials and thermogenesis in a mitochondrial respiratory chain. (a) Calculated overpotential in each reaction at respiration activity of 4 e^−^ per site per s for NADH oxidation in complex I, succinate oxidation in complex II, ubiquinone reduction in complex I and II, ubiquinol oxidation in complex III, cytochrome *c* reduction in complex III, cytochrome *c* oxidation in complex IV, and oxygen reduction reaction (ORR) in complex IV. Total is the summation of all the overpotential in each reaction, and it is corresponded to overall heat, (b) estimated total overpotential and thermogenesis ratio applied in a respiratory chain as a function of respiration activity. The red part is the ratio of thermogenesis derived from overpotential, and blue part is the remaining energy that can be used to pump the proton from matrix to intermembrane for ATP synthesis or thermogenesis by proton leak. The range of non-UCP1 cell and UCP1 cell around 0.5–7 e^−^ per site per s and 7–25 e^−^ per site per s respectively. The range was estimated by interquartile range of data in Fig. S20(a). The horizontal reference line at 1.04 V represents the flux-weighted total driving force (Δ*E*^0^_eff_), calculated as a weighted average of 80% × 1.1 V (NADH/CI route) and 20% × 0.8 V (succinate/CII route).

**Fig. 3:**
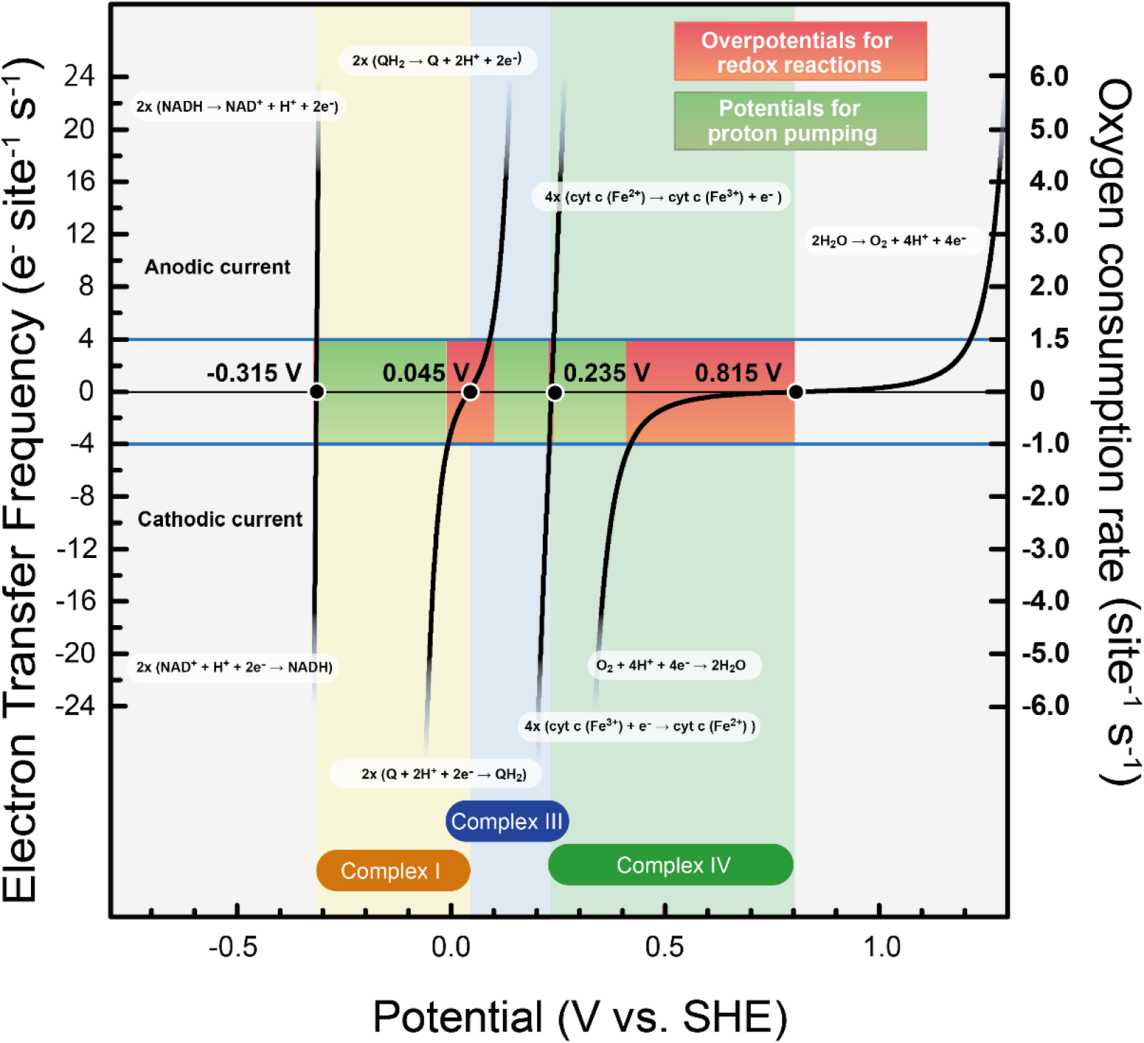
Polarization curves of each enzymatic reaction in complex I, III, and IV based on kinetic parameters estimated in the present study. The pink and light green areas correspond to the power used for overpotential followed by heat dissipation and for proton pumping for ATP synthesis, respectively.

In this calculation, we assumed that 80% of electrons enter the respiratory chain through complex I and 20% through complex II, after which both pathways converge at complex III as observed in physiological respiration. For system-level energy accounting in Fig. 2b, we used the flux-weighted driving force Δ*E*^0^_eff_ = 0.8 × 1.1 V + 0.2 × 0.8 V = 1.04 V. Although this ratio can shift in proliferating cells or under other physiological conditions,^43–45^ we performed sensitivity analysis to demonstrate the generality of our model (see Fig. S26). Fig. 3 analyzes the NADH branch only (reference Δ*E*^0^ = 1.1 V) to isolate CI → CIII → CIV partitioning.

### Estimated overpotential and thermogenesis in a mitochondrial respiratory chain

Using the method described above, the exchange current densities (*j*_0_) for each enzymatic redox step were calculated and summarized in Table S3. The results show that the ORR in complex IV has a markedly lower *j*_0_ (5.1 × 10^−11^ to 1.1 × 10^−8^ A cm^−2^) than the reactions in complexes I–III (approximately 4.1 × 10^−6^ to 7.5 × 10^−2^ A cm^−2^). Because *j*_0_ reflects the intrinsic electron-transfer rate at equilibrium, a smaller *j*_0_ indicates slower kinetics. Thus, the comparatively low *j*_0_ of complex IV quantitatively supports the conclusion that ORR is the kinetically limiting step and the dominant contributor to the overall overpotential in the respiratory chain.

To quantify both the overpotential and the resulting heat production for each enzymatic step in the mitochondrial respiratory chain, we evaluated complexes I, III, and IV under physiological electron transfer rates. Fig. 2a shows the calculated overpotentials at a steady-state electron transfer frequency *Γ*_e_ of 4 e^−^ per site per s for each reaction in complexes I, II, III, and IV, together with the total overpotential. The total overpotential was calculated using eqn (3) and the parameters listed in Table S3. A detailed explanation of the calculation of each parameter is provided in the SI, Section two. The oxygen reduction reaction (ORR) in complex IV exhibits the largest value of overpotential, 0.54 ± 0.10 V, whereas ubiquinone reduction in complex I and ubiquinol oxidation in complex III show much smaller overpotentials of 0.04 ± 0.02 V. Succinate oxidation in complex II contributes ∼20% of flux but shows <0.001 V overpotential, indicating negligible heat dissipation. Because lower *Γ*_e_0__ values correspond to slower intrinsic activity, these results indicate that kinetically sluggish steps produce larger overpotentials and therefore greater heat dissipation. The cumulative overpotential for the entire respiratory circuit is about 0.68 V, meaning that ∼65% of the total Gibbs free energy change (1.04 V) is lost as overpotential and converted into heat.

Fig. 2b plots the total overpotential against *Γ*_e_ in the range 0.5–25 e per site per s, corresponding to physiological mitochondrial respiration rates, with and without proton leak. The horizontal line at 1.04 V indicates the flux-weighted total driving force of the respiratory chain Δ*E*^0^_eff_, reflecting ∼80% contribution from NADH oxidation (CI, 1.1 V) and ∼20% from succinate oxidation (CII, 0.8 V), both coupled with the ORR. The data reveal how this energy is partitioned between ATP synthesis and heat production: as *Γ*_e_ increases, the total overpotential rises non-linearly, leading to a higher fraction of the energy being released as heat. To validate our model for physiological condition, we compare the *Γ*_e_ from in vivo and *in vitro* study in Fig. S19. It shows that the average between *Γ*_e_ of *in vivo* and *in vitro* study is not significantly different. Therefore, this range of *Γ*_e_ that we choose is relevant for physiological condition.

Quantitatively, the model predicts that thermogenesis accounts for 46–72% of the total energy yield across the physiological range of *Γ*_e_ without proton leak, with uncertainties of approximately ±20% on the minimum and maximum values. This range is consistent with literature estimates of 40–60%^46,47^ and, together with comparisons across different cell types (See Fig. S2), supports the validity of the overpotential-derived thermogenesis model. Fig. S2 shows that immune cells or blood-derived cells characterized by intermittent activity, exhibit an average of thermogenesis ratio approximately 41%, whereas cells originating from highly active organs such as heart, liver, and kidney have average ratios approximately 68%. This result is consistent with the established understanding that metabolically active organs such a liver, kidney, and heart serve as the predominant contributor to basal metabolic rate (BMR) in human body.^48,49^ The analysis further shows that more than 70% of this heat originates from complex IV, highlighting it as the primary thermogenic site in the respiratory chain. Although complex II supplies ∼20% of electron input, its negligible overpotential renders its thermogenic contribution minor. It should be noted that the schematic analysis in Fig. 2 assumes a 1 : 1 : 1 stoichiometry among complexes I + II, III, and IV for simplicity. In reality, the copy number of each complex may differ, and for example, if complex IV is less abundant, the per-site heat dissipation would increase proportionally. However, the total contribution from complex IV to the overall thermogenesis remains invariant, since the product of per-site dissipation and copy number is conserved. This consideration emphasizes that the conclusion regarding complex IV as the dominant thermogenic site is general and not dependent on the assumed stoichiometry. Finally, the present analysis shows that mitochondrial heat production can be quantitatively explained by reaction overpotentials along the respiratory chain, without the need to explicitly invoke heat generation originating from proton pumping itself. This implies that proton-motive energy is predominantly conserved for productive processes such as ATP synthesis or proton leak, rather than being dissipated as an independent heat source.

Importantly, experimental observations that proton-leak uncoupling accelerates respiration can be rationalized within the present framework as follows. Proton leak lowers the membrane potential, thereby reducing the energetic cost required for proton pumping against the electrochemical gradient. As a consequence, a larger fraction of the available electrochemical driving force is expressed as reaction overpotential rather than being expended on proton pumping work. This redistribution increases the reaction overpotential at each enzymatic redox step, accelerates electron transfer, and directly enhances heat dissipation through overpotential losses (see Fig. S27).

Within this framework, mitochondrial heat production is quantitatively governed by energy dissipation associated with reaction overpotentials, characterized by the product of overpotential and electron-transfer frequency (*η* × *Γ*_e_). Proton leak therefore modulates the membrane potential and shifts the energetic balance toward overpotential dissipation along the respiratory chain, leading to increased *Γ*_e_ and amplified overpotential-driven heat generation.

For clarity, the mechanistic sequence underlying classical uncoupling phenomena can be summarized as follows: proton leak → reduced membrane potential → decreased energetic cost of proton pumping → increased reaction overpotential (*η*) → increased electron-transfer frequency (*Γ*_e_) → enhanced heat dissipation. This physical picture naturally accounts for well-established thermogenic behavior associated with proton leak. In particular, UCP1-expressing brown adipose tissue exhibits *Γ*_e_ values nearly fivefold higher than those of non-UCP1 cells (see Fig. S20), and the present model predicts corresponding thermogenesis efficiencies of 70–90% (Fig. 2b), consistent with their established role as dedicated thermogenic tissues. Under these conditions, proton leak does not constitute a separate or competing heat-generation mechanism but rather defines a physiological regime in which overpotential-driven dissipation is strongly amplified.

### Energy partitioning between ATP synthesis and heat production

To determine how the energy from mitochondrial respiration is divided between ATP synthesis and heat production, we analyzed the contributions of each complex using the results in Fig. 2. In our analysis, the energy for ATP synthesis was assumed to be equivalent to the energy required for proton pumping. Specifically, the energy available for proton pumping is determined by subtracting the calculated overpotential from the total available thermodynamic driving force. This approach assumes that the energy partitioning is predominantly governed by the balance between overpotential-driven heat dissipation and proton pumping work. Fig. 3 presents the polarization curves for each enzymatic reaction within complexes I, III, and IV, plotted against potential. Positive currents correspond to oxidation reactions occurring at the anode, while negative currents correspond to reduction reactions at the cathode; under steady-state conditions, these currents have equal magnitudes but opposite signs. For example, in complex I, NADH → NAD^+^ + H^+^ + 2e^−^ represents the anodic reaction, whereas Q + 2H^+^ + 2e^−^ → QH_2_ corresponds to the cathodic reaction.

In Fig. 3, we illustrate the case of *Γ*_e_ = 4 e^−^ per site per s as an example focusing on the NADH branch only (CI → CIII → CIV; Δ*E*^0^ = 1.1 V), since the succinate/CII pathway contributes little to heat dissipation and is shown separately in Fig. 2. The pink-shaded area indicates the energy dissipated as heat from overpotential, while the yellow-green area corresponds to the energy used for proton pumping in ATP synthesis. At this *Γ*_e_ value, the ratio of the pink area to the combined pink and yellow-green areas is 53%, indicating a heat production fraction of 53%, consistent with the result in Fig. 2b.

A key observation from Fig. 3 is that complexes I and III primarily direct their energy to ATP synthesis (*i.e.*, proton pumping), whereas complex IV allocates more than half of its energy to heat generation. Heat production in complexes I and III accounts for only ∼20% of their driving force, but in complex IV, over 80% of the input energy is dissipated as heat, suggesting a functional localization of thermogenesis within the respiratory chain.

The present analysis shows that the number of protons pumped per electron, estimated from overpotential-based energy partitioning, agrees well with proton stoichiometries established by biochemical studies. From these polarization curves, the number of protons pumped by each respiratory complex can be estimated by dividing the remaining available energy by the energetic cost of proton translocation. Using an established transmembrane potential difference for proton transfer of 0.15 V,^50^ the energy allocated to proton pumping (yellow-green area) is approximately 0.30–0.34 eV, 0.15–0.18 eV, and 0.03–0.12 eV for complexes I, III, and IV, respectively. Assuming that the energetic cost of translocating one proton across the membrane is 0.15 eV, these values correspond to ∼2.0–2.3, 1.0–1.2, and 0.2–0.8 protons pumped per electron transferred. Because the oxygen reduction reaction (ORR), O_2_ + 4H^+^ + 4e^−^ → 2H_2_O, involves the transfer of four electrons per oxygen molecule, the corresponding proton numbers amount to 8–9.2, 4–4.8, and 0.8–3.2 protons per O_2_ for complexes I, III, and IV, respectively. Notably, the protons formally attributed to complex III arise from the quinone-mediated redox loop, which couples quinone reduction upstream of complex III and quinol oxidation at complex III itself. Under ideal coupling conditions, this redox loop contributes up to 8 protons per O_2_ molecule.^51,52^ In addition, complex I independently pumps protons through its membrane-embedded antiporter-like subunits, contributing a further 8 protons per O_2_ in the canonical biochemical scheme, whereas complex IV contributes an additional 4 protons per O_2_ molecule *via* redox-coupled proton pumping tightly associated with O_2_ reduction.^51,52^ These values represent theoretical upper limits assuming full utilization of the available electrochemical driving force for proton translocation. In contrast, the present analysis estimates the effective number of protons pumped under physiological conditions based on overpotential-derived energy partitioning. For a transmembrane potential of 0.15 V, the total number of protons effectively pumped across the respiratory chain is estimated to be approximately 13–18 per O_2_ molecule. This range reflects partial utilization of the available driving force, as well as the fact that electrons entering *via* complex II do not contribute to proton pumping at complex I. When this effect is taken into account, the expected upper bound corresponds to (8 × 0.8 + 8) + 4 ≈ 18.4 protons per O_2_, where the factor 0.8 accounts for the fractional electron flux bypassing proton pumping at complex I, and the additional 4 protons originate from the downstream redox loop associated with complex III and IV. The resulting estimate is fully consistent with established stoichiometries of mitochondrial proton translocation when overpotential-dependent energy dissipation is considered.^53,54^

Biochemical studies have consistently shown that proton pumping at complex IV is intrinsically limited to approximately one proton per electron,^55^ substantially smaller than the redox energy available at complex IV, indicating that the remaining energy is expended as overpotential, in agreement with the present analysis. As illustrated in Fig. 3, electron transfer from complex III to complex IV spans a large redox energy window: electrons are supplied from cytochrome *c* at approximately 0.235 V, whereas the equilibrium potential for the oxygen reduction reaction (ORR) at complex IV is ∼0.815 V, providing ∼0.58–0.60 V per electron for energy conversion. Because proton pumping at complex IV can store energy only up to the transmembrane potential (∼0.15 V per proton), the remaining energy is necessarily consumed as net overpotential.

This energetic imbalance—large available redox energy but intrinsically limited proton-pumping capacity—therefore constitutes direct quantitative evidence that complex IV operates in an overpotential-dominated regime. The present analysis explicitly identifies this unavoidable overpotential as the primary origin of heat generation at complex IV.

Based on Fig. 2b, the thermogenesis fraction derived from overpotential dissipation is estimated to be approximately 50–60% under coupled respiration conditions without UCP-1, and increases to ∼90% under UCP-1 – mediated uncoupled respiration. When converted to heat production per oxygen molecule, these values correspond to ∼210–250 kJ per mol O_2_ and ∼380 kJ per mol O_2_, respectively. Notably, these estimates agree well with calorimetric measurements reported for isolated mitochondria, where the heat released per oxygen consumed has been determined to be 224 ± 22 kJ per mol O_2_ under coupled conditions and ∼440 kJ per mol O_2_ under chemically uncoupled states. This quantitative agreement demonstrates that the present overpotential-based framework accurately captures the thermogenic dynamics of mitochondrial respiration.^56^

It should also be noted that the membrane potential is not constant but varies with the respiratory rate, which in turn affects the effective number of protons pumped per electron. As shown in Fig. S24, a higher membrane potential corresponds to a fewer number of protons pumped, whereas a lower membrane potential results in larger protons pumped, consistent with trends reported in previous studies.^57^ We further analyzed the dependence of proton pumping on the electron-transfer frequency *Γ*_e_. As shown in Fig. S25, at low *Γ*_e_, corresponding to resting or low-activity conditions, the number of protons pumped from the matrix to the intermembrane space is relatively high. As *Γ*_e_ increases, the number of protons pumped per electron decreases. This behavior is fully consistent with the present overpotential-based framework. At low *Γ*_e_, reaction overpotentials are small and the membrane potential remains high, allowing a larger fraction of the available electrochemical driving force to be utilized for proton pumping and ATP synthesis. In contrast, at high *Γ*_e_, the increased reaction overpotentials required to sustain rapid electron transport reduce the fraction of energy available for proton pumping, leading to a decrease in the effective proton stoichiometry.^58–60^ Consequently, the present model provides a unified and quantitative explanation for the established relationship between proton pumping efficiency, membrane potential, and oxygen consumption rate in mitochondrial bioenergetics.

### Chemical physics implications

The originality of this study lies in introducing the electron transfer frequency (ETF) as a kinetic descriptor to quantitatively predict heat-generating energy dissipation in biological electron-transfer chains through overpotential analysis, a framework long established in electrocatalysis. This approach is enabled by the decades of electrochemical research on enzymatic reactions. The presence of overpotential reflects the activation barrier of the reaction, with slower reaction kinetics corresponding to lower electron-transfer rates. In an electrical circuit analogy, the overpotential plays a role analogous to electrical resistance, leading to energy dissipation as Joule heat under steady-state current flow. In the present work, we adopted a macroscopic description based on a simplified electrical circuit model, as schematically illustrated in Fig. 1d. In reality, however, a more refined circuit representation would need to explicitly account for the number, spatial organization, and coupling of enzymes within each respiratory complex. Such a description would likely involve incorporating complex subcircuits that dynamically regulate local potentials and electron-transfer pathways. Elucidating these more detailed mechanisms remains an important subject for future investigation. In addition, integrating explicit proton-pumping mechanisms into the overpotential-based electrochemical framework represents another key challenge for future studies.

A fundamental academic contribution of this study is to place biological respiration within the framework of steady-state-nonequilibrium thermodynamics, and to describe its energetics using an electrochemical formulation. Unlike classical equilibrium thermodynamics—where maximum efficiency is achieved only in infinitely slow, reversible processes that yield zero net power—living systems must operate continuously at finite reaction rates. Under such steady-state conditions, a finite driving force (overpotential) is inevitably required to sustain electron transfer, leading to irreversible energy dissipation as heat. From a theoretical perspective, the system is governed by the steady-state-nonequilibrium thermodynamics originally formulated by Prigogine, in which energy conversion is governed not only by thermodynamic state functions but by the pathways through which energy is transformed and dissipated.^16,61^ In this framework, the rate of entropy production within the system is balanced by the rate of entropy dissipation to the surroundings under steady-state conditions, as detailed in the SI. Importantly, in steady-state electrochemical reactions, the Gibbs free energy difference—the fundamental thermodynamic driving force—does not dissipate directly as heat. Instead, it is first converted into a nonequilibrium thermodynamic force, namely the overpotential, which accelerates reaction kinetics. This overpotential is then irreversibly dissipated as heat through sustained current flow. As we have previously reported, this dissipation manifests as Joule heat, given by the product of current and overpotential under steady-state conditions (see SI). Consequently, heat production is an intrinsic feature of the living state sustained by respiration, and it ceases when respiration stops and life ends. This principle is formally analogous to that of an electrical resistive heater, in which heat is generated only while current flows. By describing biological respiration in terms of electrochemical energy conversion, overpotential generation, and steady-state dissipation, this study represents an initial step toward an electrochemical interpretation of bioenergetics and provides a complementary perspective for understanding energy conversion and thermogenesis in living systems.

## Conclusions

In this study, we established that mitochondrial heat production can be quantitatively understood as a consequence of overpotentials arising at enzymatic redox steps—a thermodynamic phenomenon long recognized in electrocatalysis. While the free energy difference between complexes I and IV in the respiratory chain is partially conserved for ATP synthesis, the remaining energy is irreversibly dissipated as heat to sustain finite reaction rates. Using experimentally measured electrochemical parameters, we estimated steady-state overpotentials for complexes I, III, and IV, and demonstrated that the total energy dissipation accounts for approximately 46–72% of the respiration-driven Gibbs free energy, in agreement with calorimetric data. This model also correctly reproduces the proton flux expected from ATP synthesis, reinforcing its consistency with classical chemiosmotic mechanisms. Importantly, our analysis identifies the oxygen reduction reaction (ORR) in complex IV as the dominant thermogenic site in the respiratory chain, as its multiple elementary steps render the reaction sluggish, which in turn require a large overpotential. Succinate/CII, despite providing ∼20% of flux, functions as a low-*η* parallel entry and contributes negligibly to heat generation. This conclusion is general and remains valid even when considering non-uniform copy numbers of respiratory complexes, since per-site dissipation scales inversely with abundance while the total contribution is conserved. Thus, the insight refines our understanding of where and how energy dissipation occurs during aerobic respiration. By framing mitochondrial thermogenesis within a physical chemistry perspective, this work bridges electrocatalysis and bioenergetics, and provides a framework for future investigations into metabolic regulation and mitochondrial dysfunction in health and disease. In particular, the introduction of electron transfer frequency (ETF) as a site-specific descriptor—directly analogous to turnover frequency (TOF) in heterogeneous catalysis—provides a new quantitative basis for analyzing enzymatic redox kinetics. Beyond mitochondria, the present framework illustrates how quantitative overpotential analysis, widely applied in electrochemical catalysis, can be generalized to enzymatic systems, bridging the gap between chemistry and biology in the study of energy dissipation.

## Author contributions

K. T. and J. N. conceived the quantitative analysis of mitochondrial overpotentials. K. T. designed an electrochemical circuit model for the respiratory chain. J. N. conceived thermogenesis by overpotential. J. N. and K. T. designed analytical protocols. N. A. P. N. performed the analyses and the calculations. N. A. P. N., M. Y., J. N. and K. T. discussed the results and prepared the manuscript for publication.

## Conflicts of interest

There are no conflicts to declare.

## Supplementary Material

SC-OLF-D5SC06693J-s001

## Data Availability

The data supporting the findings of this study are available within the article and its supplementary information (SI). Additional data and analysis details are available from the corresponding author upon reasonable request. Supplementary information is available. See DOI: https://doi.org/10.1039/d5sc06693j.
