# Supplementary material for: Enzymatic oxygen reduction dominates overpotential-driven thermogenesis in mitochondria
Source: Chem Sci. 2026 Mar 30;17(25):12512–21. doi: 10.1039/d5sc06693j (PMC13182749; doi:10.1039/d5sc06693j)
Supplement: SC-017-D5SC06693J-s001 [file SC-017-D5SC06693J-s001.pdf]

Supplementary Information (SI) for Chemical Science.
This journal is © The Royal Society of Chemistry 2026

Supplementary Information for

Enzymatic oxygen reduction dominates overpotential-driven thermogenesis in mitochondria

Authors
Nuning Anugrah Putri Namari[a,d], Mo Yan[a,d], Junji Nakamura*[a,b,c], Kotaro Takeyasu*[b,c,e]

Affiliations

[a] International Institute for Carbon-Neutral Energy Research (I^2CNER), Kyushu University; 744 Motooka, Nishi-ku, Fukuoka-shi, Fukuoka 819-0395, Japan.

[b] Department of Materials Science, Institute of Pure and Applied Sciences, University of Tsukuba; 1-1-1 Tennodai, Tsukuba, Ibaraki 305-8573, Japan.

[c] Tsukuba Research Center for Energy and Materials Science, University of Tsukuba; 1-1-1 Tennodai, Tsukuba, Ibaraki 305-8573, Japan.

[d] Graduate School of Science and Technology, University of Tsukuba; 1-1-1 Tennodai, Tsukuba, Ibaraki 305-8573, Japan.

[e] Institute for Catalysis, Hokkaido University; N21W10, Sapporo, Hokkaido 001-0021, Japan.

*Correspondence to: takeyasu@cat.hokudai.ac.jp (K.T.)
and nakamura.junji.700@m.kyushu-u.ac.jp (J.N.)

TABLE OF CONTENTS

S1. ESTIMATION OF ELECTRON TRANSFER FREQUENCY (Γ_E) IN MITOCHONDRIA **S3**

S2. ESTIMATION OF OVERPOTENTIAL **S9**

S2-1 ANALYSIS OF CYCLIC VOLTAMMETRY (CV) DATA TO OBTAIN EXCHANGE CURRENT DENSITY (J_0)....S9

(1) NADH Oxidation..S10

(2) Succinate Oxidation Data 1..S11

(3) Succinate Oxidation Data 2..S12

(4) Ubiquinone Reduction and Oxidation Data 1..S13

(5) Ubiquinone Reduction and Oxidation Data 2..S15

(6) Ubiquinone Reduction and Oxidation Data 3..S16

(7) Cytochrome c Reduction and Oxidation Data 1..S17

(8) Cytochrome c Reduction and Oxidation Data 2..S18

S2-2 ANALYSIS OF LINEAR SWEEP VOLTAMMETRY (LSV) DATA TO OBTAIN EXCHANGE CURRENT DENSITY (J_0) S20

(1) ORR data 1..S20

(2) ORR data 2..S21

(3) ORR data 3..S21

(4) ORR data 4..S22

(5) ORR data 5..S23

(6) ORR data 6..S24

(7) ORR data 7..S25

S3. ESTIMATION OF THE THERMOGENESIS RATIO AT DIFFERENT ELECTRON TRANSFER FREQUENCY (Γ_E) **S26**

S5. ETF OF PROTON LEAK **S28**

S6. SENSITIVITY ANALYSIS **S30**

.... **S32**

S7. HEAT VS ETF **S33**

S8. OVERPOTENTIAL-DRIVEN THERMOGENESIS BASED ON NON-EQUILIBRIUM THERMODYNAMICS **S34**

REFERENCES: **S36**

S1. Estimation of electron transfer frequency (Γ_e) in mitochondria

As mentioned in the main text, we introduced steady-state electron transfer frequency (Γ_e), defined as the number of electron transfers per active site per second (e^- site^{-1} s^{-1}) to account for the multiple sequential electron transfer reactions occurring at enzyme active sites within the mitochondrial respiratory chain. Considering the number of active sites is crucial for kinetic analysis of enzymatic electrochemistry. Therefore, Γ_e represents the number of electron transfers through a single active site per second. Γ_e was estimated from the oxygen consumption rate (OCR) of different cell types[1] and the number of complex IV proteins within the cells, as listed in Table S1. The average number of complex IV molecules was calculated, and the estimated number of complex IV proteins per mg of protein is shown in Table S2. Notably, heart cells contain the highest amount of complex IV proteins compared to other cell types. Consequently, an average value of approximately 4.5×10^{14} sites per mg of protein was used for heart cells, whereas a value of 1.0×10^{14} sites per mg of protein was assumed for other cell types. Sensitivity analysis was conducted (see Fig S23) for the rationality of choosing this parameter. This Γ_e was then used to calculate realistic overpotentials for each complex within the mitochondrial respiratory chain.

An example of the steps to estimate Γ_e is shown below:

Step 1. Find the OCR data

Consider the example of a Rat hepatocyte cell in Table S1(No 25), which has an OCR of 350×10^{-18} mol s^{-1} ng-protein^{-1}

$$\mathrm{OCR} = 350 \times 10^{-18} \text{ mol s}^{-1} \text{ ng-protein}^{-1} = 350 \times 10^{-12} \text{ mol s}^{-1} \text{ mg-protein}^{-1}$$

Step 2 Estimate the OCR per site

Using the value of complex IV at 1.0×10^{14} sites per mg of protein and Avogadro's number (6.022×10^{23} molecules mol^{-1}), convert the OCR to the number of oxygen molecules consumed by mitochondria per second (O_2 molecules s^{-1} site^{-1}).

$$\text{OCR per site} = \frac{350 \times 10^{-12} \text{mol s}^{-1} \text{ mg-protein}^{-1} \times (6.022 \times 10^{23} \text{ molecules mol}^{-1})}{1.0 \times 10^{14} \text{ site mg-protein}^{-1}}$$
$$= 2.1 \text{ O}_2\text{-molecules site}^{-1}\text{s}^{-1}$$

Step 3. Convert OCR to Γ_e

In the electron transport chain (ETC), four electrons are required to convert one oxygen molecule into two water molecules. Therefore, Γ_e is estimated as:

$$\Gamma_e = 2.1 \text{ O}_2\text{-molecules site}^{-1}\text{s}^{-1} \times 4 \text{ e}^- \text{ O}_2\text{-molecules}^{-1} = 8.4 \text{ e}^- \text{ site}^{-1}\text{s}^{-1}$$

This is how the number of Γ_e with unit e^- site^{-1} s^{-1} was determined. Using this procedure, all the OCR data in Table S1 were converted to Γ_e, and the estimated numbers of Γ_e are shown in Fig S1. Except for the MCF-7 cell (breast cancer cells), the other cells had Γ_e ranging from approximately 0.15 to 10 e^- site^{-1} s^{-1}. Therefore, this range was used to calculate the overpotential. As shown in Table S1, the units of OCR were 10^{-18} mol s^{-1} ng-protein^{-1}. Some data are presented in units of 10^{-18} mol cell^{-1} s^{-1} (see in the Ref[1]). Given that the mean weight of a cell is 1 ng[2], the unit of 10^{-18} mol s^{-1} ng-protein^{-1} was used for OCR.

Table S1. Summary of literature-reported oxygen consumption rates (OCR) of various cells, including cells names, and cell types used in this study

NO	Cell Name	Cell Type	OCR (mol s^{-1} mg-protein^{-1})	Ref
1	AFP-27	Murine Hybridoma Cell	6.0E-12	1
2	J774A.1	Murine Macrophages	6.2E-12	
3	MEF	Mouse Embryonic Fibroblasts	7.0E-12	
4	EL4	Murine T cell lymphomas	7.7E-12	
5	BW1100	Murine Mastocytoma Cell	8.1E-12	
6	Rat Neutrophils	Rat Neutrophils	8.3E-12	
7	RAW264.7	Transformed Mouse macrophages	8.9E-12	
8	A20	Mature Murine B cell lymphoma	1.0E-11	
9	BM MNC	Human Bone Marrow	1.1E-11	
10	D2SC/1	Murine Dendritic cell line	1.3E-11	
11	AG08472	Vascular endothelial cells of the pig	1.7E-11	
12	TIME Cells	Endothelial cells	2.8E-11	
13	Neural Stem cells	Murine Neural Stem Cells	3.1E-11	
14	Embryonic Stemcell	Murine Embryonic Stemcell	4.0E-11	
15	NRVM	Rat Ventricular myocyte	4.0E-11	
16	AG08473	Pig Thoracic Aorta	4.4E-11	
17	Hybridoma	Murine Hybridoma Cell	6.1E-11	
18	Synaptosomes	Rat Brain	6.5E-11	
19	Bovine endothelial	Aortrae of Cattle	6.7E-11	
20	BHK	Baby Hamster Kidney	8.3E-11	
21	Podocytes	Kidney cell	8.3E-11	
22	CHO	Chinese Hamster Ovary cells	8.6E-11	
23	Myocytes	Neonatal cardiomyocytes	1.0E-10	
24	MAF-E	Adult Fallopian Tube	1.1E-10	
25	HepG2	Human Hepatoma cells	1.1E-10	
26	Renal Mesangial	Rat kidney cell	1.5E-10	
27	Hep3B	Human Hepatoma cells	1.6E-10	
28	Rat fibroblast	Rat Embryo Fibroblast	1.9E-10	
29	Heart non-muscle new	Newborn rat heart non muscle	2.0E-10	
30	L-6 myoblast	Human Muscle	2.0E-10	
31	Porcine hepatocytes	Porcine hepatocytes Day 4	3.0E-10	
32	LLC-PK	Pig Kidney	3.2E-10	
33	Rat hepatocytes	Rat hepatocytes	3.5E-10	
34	LLC-MK	Rhesus monkey kidney	4.7E-10	
35	Beating cardiac	Old rats cardiac myocytes	1.2E-09	
36	Cardiomycocyte	Heart cell	5.8E-09	3

37	Cardiomycocyte	Heart cell	3.0E-09	
38	Liver Crocodile	Liver	1.8E-10	4
39	Liver Rat	Liver	3.3E-10	
40	Liver Duckling	Liver	5.0E-10	
41	Differentiated Fat cells	Fat Cells	1.7E-10	5
42	Proximal tubular	Kidney cell	1.3E-10	6
43	Glomeruli	Kidney cell	6.3E-10	
44	Plant mitochondria	Plant	3.3E-10	7
45	Liver	Liver	3.3E-10	8
46	Kidney	Kidney cell	1.5E-09	
47	Human Fibroblast	Fibroblast	7.0E-12	9
48	Blood	Blood	1.3E-10	10
49	Rat Heart	Heart cell	1.3E-09	11
50	Mouse liver	Liver cell	6.7E-10	
51	Cultured astrocytes	Brain Cell	6.7E-11	12
52	Cultured astrocytes	Brain Cell	5.0E-11	

Table S2. Summary of complex IV (CIV) content and calculated number of CIV per mg protein with measurement method and literature source.

No	Name	CIV content, the original unit from reference	Number of CIV per mg protein	Method	Ref
1	Bovine Heart	0.6–1.0 nmol mg-protein^{-1}	$3.6 \times 10^{14} – 6.022 \times 10^{14}$	Spectrophotometry	13
2		0.8–1.13 nmol mg-protein^{-1}	$4.82 \times 10^{14} – 6.80 \times 10^{14}$		14
3		1.31 nmol mg-protein^{-1}	7.89×10^{14}		15
4	Beef Heart	0.6 nmol mg-protein^{-1}	3.6×10^{14}	Spectrophotometry	16
5		0.33–0.5 μmol g-protein^{-1}	$1.9 \times 10^{14} – 3 \times 10^{14}$	Spectrophotometry	17
6	Pig Heart	0.84 nmol mg-protein^{-1}	5.05×10^{14}	Spectrophotometry	18
7	Rat Heart	0.32 nmol mg-protein^{-1}	1.93×10^{14}	Spectrophotometry	19
8	Rat Muscle	0.277 nmol mg-protein^{-1}	1.37×10^{14}	Spectrophotometry	19
9	Rat Liver	0.077 nmol mg-protein^{-1}	4.64×10^{13}	Spectrophotometry	20
10		0.222 nmol mg-protein^{-1}	1.3×10^{14}	Steorogical using electron microscope	21
11		0.095 nmol mg-protein^{-1}	5.72×10^{13}	Spectrophotometry	22
12		0.093 nmol mg-protein^{-1}	5.60×10^{13}	Spectrophotometry	19
13		0.243 nmol mg-protein^{-1}	1.46×10^{14}	Spectrophotometry	23
14	Rat kidney	0.119 nmol mg-protein^{-1}	7.17×10^{13}	Spectrophotometry	19
15	Rat Brain	0.125 nmol mg-protein^{-1}	7.53×10^{13}	Spectrophotometry	19

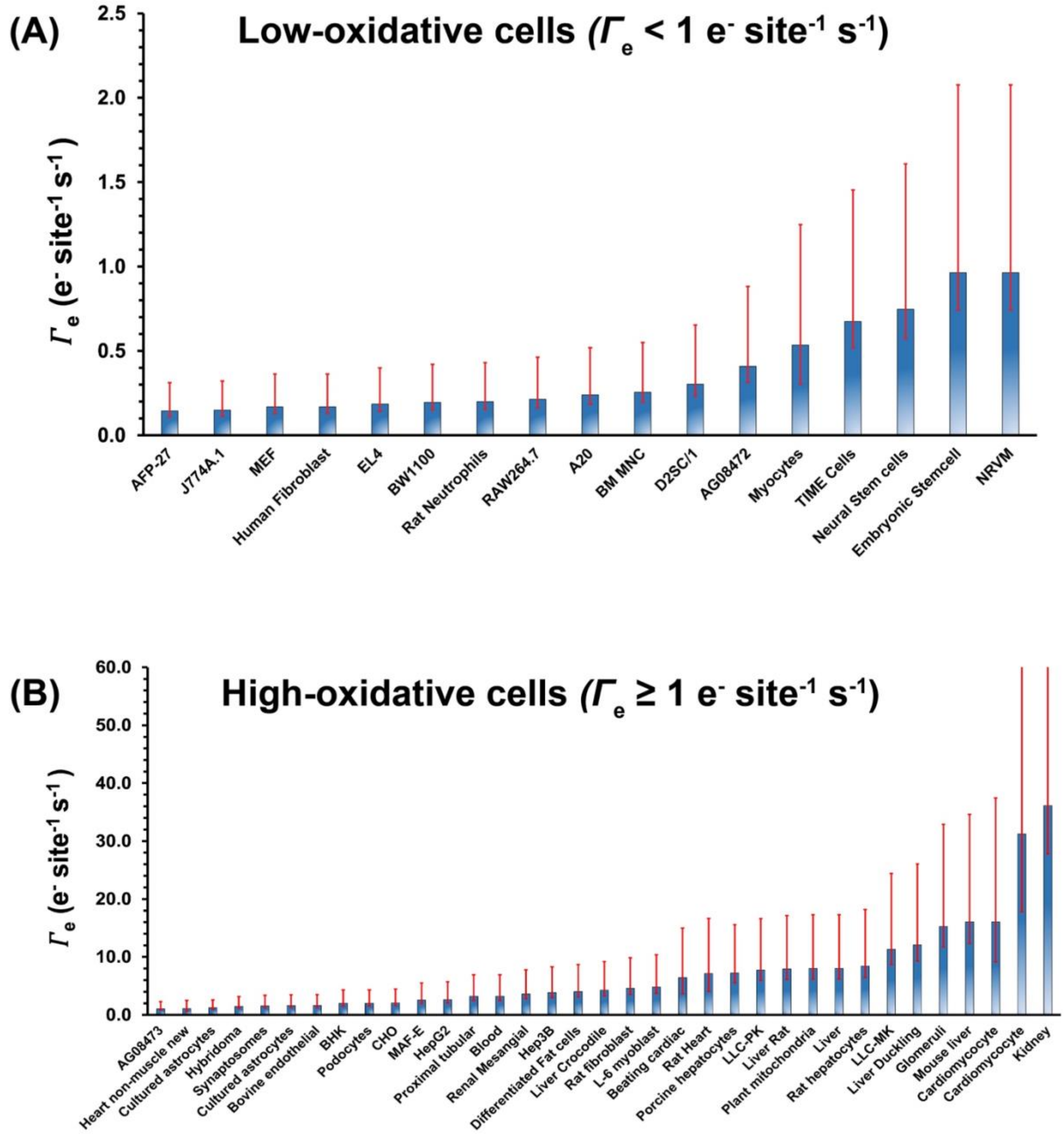

Figure S1. Estimated Γ_e values for the cell types listed in Table S1. (A) Low-oxidative cells ($\Gamma_e < 1$ e^- $site^{-1}s^{-1}$), including immune cells, stem cells, fibroblast, and neonatal cardiomyocytes. (B) High-oxidative cells with ($\Gamma_e \geq 1$ e^- $site^{-1}$ s^{-1}), including metabolically demanding tissues such as kidney, brain, liver, and heart cells.

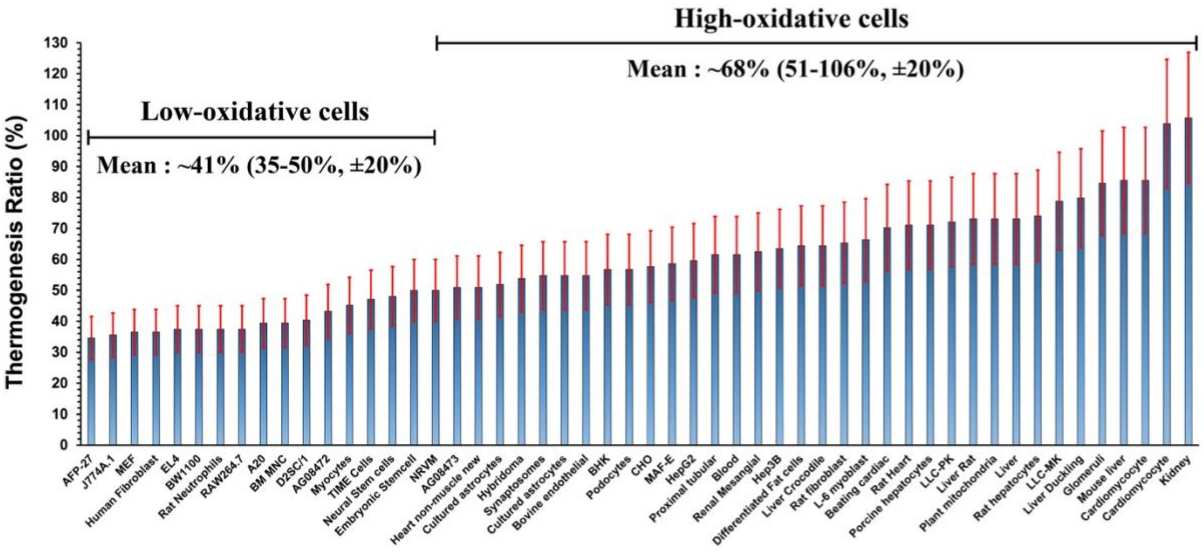

Figure S2. Calculated thermogenesis ratios for the cell types shown in Figure S1. Estimated Γe values for the cell types listed in Table S1. (A) Low-oxidative cells (Γe < 1 e- site-1s-1), including immune cells, stem cells, fibroblast, and neonatal cardiomyocytes. (B) High-oxidative cells with (Γe ≥ 1 e- site-1 s-1), including metabolically demanding tissues such as kidney, brain, liver, and heart cells. . Low-oxidative cells exhibit a mean thermogenesis ratio of approximately 41%, with values falling within 35-50% and an estimated uncertainty of ± 20%. High-oxidative cells shows a mean thermogenesis ratio of approximately 68% with values ranging from 51-106% and a similar uncertainty of 20%.

S2. Estimation of overpotential

All data for calculating the overpotential in each reaction in the respiratory chain were obtained from the various papers listed in Table S3. The data can be obtained as a Linear Sweep Voltammogram (LSV) or Cyclic Voltammogram (CV). The procedure for calculating the LSV and CV data is described in the following section.

Table S3. Summary of calculated exchange current density (j_0), exchange electron transfer frequency (Γ_{e0}), and $\alpha zF/RT$, together with enzyme surface density (σ) values taken from the literature; all values are used for the overpotential calculation in this study.

Reaction	Data	j_0 (A cm^{-2})	σ (mol cm^{-2})	Γ_{e0} (e$^-$ site^{-1} s^{-1})	$\alpha zF/RT$	Ref
**NADH Oxidation in Complex I** NADH ⇌ NAD^+ + H^+ + 2e^-	1	1.9×10^{-6}	7.6×10^{-13}	26.2	39.58	24
**Succinate Oxidation in Complex II** Succinate ⇌ Fumarate + 2H^+ + 2e^-	1	4.1×10^{-6}	2.0×10^{-13}	212	39.58	25
	2	1.4×10^{-5}	2.7×10^{-11}	5.28	39.58	26
**Ubiquinone Reduction in Complex I** Q + 2H^+ + 2e^- ⇌ QH_2	1	7.2×10^{-4}	4.7×10^{-8}	1.6	59.37	27
	2	2.3×10^{-3}	1.2×10^{-8}	1.9	39.58	28
	3	7.5×10^{-2}	1.5×10^{-7}	5.2	19.79	29
**Ubiquinol Oxidation in Complex III** QH_2 ⇌ Q + 2H^+ + 2e^-	1	7.2×10^{-4}	4.7×10^{-8}	1.6	59.37	27
	2	2.3×10^{-3}	1.2×10^{-8}	1.9	39.58	28
	3	6.8×10^{-2}	1.5×10^{-7}	4.7	19.79	29
**Cytochrome c Reduction in Complex III** 2Cytc (Fe^{3+}) + 2e^- ⇌ 2Cytc (Fe^{2+})	1	7.6×10^{-4}	7.1×10^{-11}	112	19.79	30
	2	2.6×10^{-2}	4.6×10^{-9}	59.6	19.79	31
**Cytochrome c Oxidation in Complex IV** 2Cytc (Fe^{2+}) ⇌ 2Cytc (Fe^{3+}) + 2e^-	1	7.6×10^{-4}	7.1×10^{-11}	112	19.79	30
	2	2.6×10^{-2}	4.6×10^{-9}	59.6	19.79	31
**Oxygen Reduction Reaction (ORR) in Complex IV** O_2 + 4H^+ + 4e^- ⇌ 2H_2O	1	5.1×10^{-11}	1.3×10^{-11}	4.1×10^{-5}	21.50	32
	2	1.1×10^{-8}	4.6×10^{-12}	2.4×10^{-2}	11.39	33
	3	2.7×10^{-10}	1.1×10^{-11}	2.6×10^{-4}	16.79	34
	4	3.5×10^{-10}	9.0×10^{-8}	4.0×10^{-8}	30.26	35
	5	5.0×10^{-10}	9.0×10^{-8}	5.8×10^{-8}	35.94	35
	6	7.5×10^{-10}	4.4×10^{-8}	1.8×10^{-7}	26.74	36
	7	8.0×10^{-9}	7.0×10^{-8}	1.2×10^{-6}	31.94	37

S2-1 Analysis of Cyclic Voltammetry (CV) data to obtain exchange current density (j_0)

The cyclic voltammetry (CV) data analysis was performed using a simulator based on the Butler-Volmer equation and Fick's Second Law of diffusion[38]. This simulator enables to fit the CV data to the experimental from the literature. Using this simulator, important kinetic parameters such as k^0 (standard rate constant) and C (Bulk concentration) were extracted from the electrochemical reaction. Initially, raw data were extracted from the references using WebPlotDigitizer[39]. Both background and experimental CV data were obtained, with background CV data representing conditions where no substance of interest is added, usually provided in the references. Then, the data were then copied into the simulator which automatically subtracts the background data from the experimental data to focus on the electrochemical behavior of the substance.

Next, the information provided by the reference, such as concentration and surface area, was input into the simulator. The simulation graph was adjusted to match the experimental graph by altering parameters such as k^0 (standard rate constant), C (bulk concentration), and D (diffusion coefficient). Matching the width of the graph can be challenging due to capacitance effects or other experimental conditions not covered by the simulator. However, it is important that the height and position of the peak closely match the experimental data to obtain accurate kinetic parameters. From this simulation, k^0 (standard rate constant, cm s^{-1})) and C_{exp} (concentration, mol cm^{-3}) were determined. Subsequently, the exchange current density (j_0, A cm^{-2}) was calculated using eq (2-1). The k^0 and j_0 are essential parameters for evaluating catalytic activity

$$j_{0\,\exp} = Fk^0 C_{exp} \tag{2-1}$$

The exchange current density ($j_{0\,\mathrm{exp}}$) above is under experimental condition, not physiological condition. Therefore, to get physiological condition, we convert the $j_{0\,\mathrm{exp}}$ to the physiological j_0 (denoted by j_0) using equation below

$$j_0 = j_{0\,\mathrm{exp}} \left(\frac{C_{\mathrm{exp}}}{C_{\mathrm{phys}}}\right)^{\alpha} \tag{2-2}$$

Where j_0, α, C_{phys}, and C_{exp} are physiological exchange current density, transfer coefficient, physiological concentration of the substrate, and experimental concentration, respectively.

Using Eq (*2-3*) below, exchange current density (j_0) was converted to Γ_e at equilibrium potential (Γ_{e0}) by dividing it by the electron charge (e, 1.6×10^{-19} C e^{-1}) and the density of enzyme (σ, mol cm^{-2}) provided in the references, converting the unit to molecules cm^{-2} using Avogadro number (N_A) :

$$\Gamma_{e0} = \frac{j_0}{e\sigma N_A} \tag{2-3}$$

Assuming α = 0.5, the overpotential for specific Γ_e was calculated using the Butler-Volmer equation below:

$$\Gamma_e = \Gamma_{e0}\left(e^{\frac{(1-\alpha)zF}{RT}\eta} - e^{-\frac{\alpha zF}{RT}\eta}\right) \tag{2-4}$$

where z is the number of electrons, F is the Faraday constant (96485 C mol^{-1}), R is the ideal gas constant (8.31 J K^{-1} mol^{-1}), and T is the temperature. An assumption of α = 0.5 was used for all reactions. Figure S22 shows that the model has low sensitivity to α, indicating that using α = 0.5 is a reasonable assumption.

Fitting procedure:
First, known parameters from reference were inputted, such as formal potential (E^{0}'), Surface area (A), concentration of bulk solution (C), Scan rate (v), and total number of electrons (n) to the simulator. Ensure this parameter fixes or does not change so much in the fitting process to ensure our result is close to the real data. Then, the fitting parameters, such as the standard rate constant (k^0), the number of electrons transferred in the rate-determining step (n_a), the stretching parameter ($\boldsymbol{\gamma}$), and second-order homogeneous rate constant (k_R) were adjusted.

The stretching parameter ($\boldsymbol{\gamma}$) in default was set to 1 which means the diffusion grid is linear. However, in some cases, the diffusion is not linear so we need to adjust it to $\boldsymbol{\gamma}$ >1 which means the diffusion grid is dense near the electrode surface or $\boldsymbol{\gamma}$ <1 which means diffusion grid is denser in the location further from the electrode surface. The second-order homogeneous rate constant (k_R) set to 0 in default. However, in some cases, the subspecies was removed from the reaction and this parameter needs to be adjusted.
Some discrepancies between the simulated curves and literature experimental curves are expected. These deviations likely originate from non-idealities that are not explicitly included in the present model, such as uncompensated solution resistance (iR drop), double layer charging, and differences in experimental protocols and conditions. In contrast, the simulations assumes idealized boundary conditions and simplified kinetic description. Therefore, a quantitative mismatch between simulation and experimental data can occur. Importantly, the kinetic parameters are extracted by fitting the key experimental features relevant to kinetics - namely the peak position, peak height, and the slope of the peak- which are repred by the model. Therefore, the extracted kinetic parameters are considered reliable within the stated assumption.

Fitting results:

(1) NADH Oxidation

In a previous study[24], the mechanism of NADH oxidation was studied using protein film voltammetry. The Fp subcomplex of complex I was adsorbed onto a Pyrolytic Graphite Electrode (PGE) (see Fig. S3(A)). The value of redox potential and active site FMN matches with FMN in complex I. Therefore, this data might be suitable to study NADH oxidation in complex I. To estimate the overpotential of NADH oxidation in complex I, the background and experimental data were extracted from Ref [24]- Fig.2 A (grey line) and B, respectively. The original raw data are shown in Fig S3(B). As mentioned above, the experimental data were subtracted from the background, and the simulation curve (Fig S3(C) – blue line) was fitted to the background-subtracted data (see Fig S3(C) – red

dotted line). The input and fitting parameters can be seen in Table S4. The concentration of the NADH used in this experiment is already in agreement with the physiological amount which is around 0.1 to 0.5 mM.[40] Therefore, $C_{exp} = C_{phys}$ so that $j_0 = j_{0\ exp}$.

In Figure S3 (C), the oxidation peak in the simulation fits the experimental data. However, several non-faradaic parts do not fit into the simulation. This may be due to the electric double layer, which was not considered in the simulation. However, it is acceptable since the peak and height of the reaction simulation are fit; therefore, we can extract the kinetic parameters.

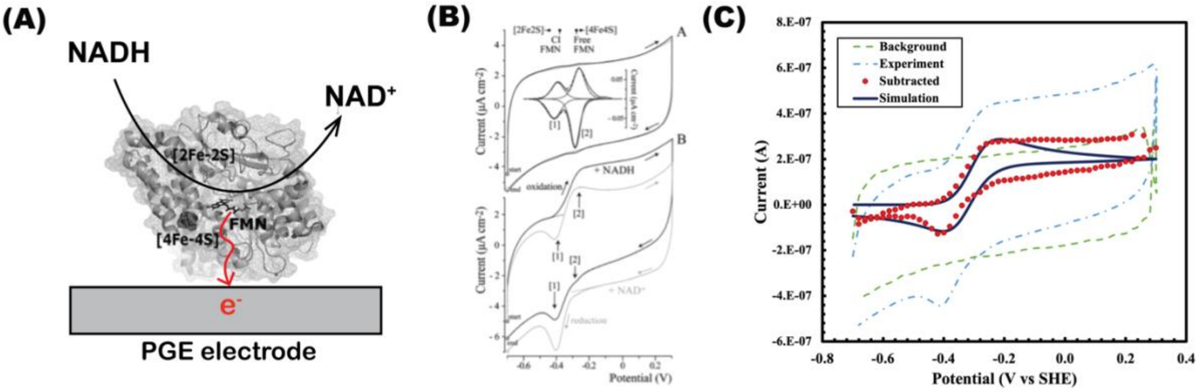

Figure S3.(A) Schematic illustration of the electrochemical system used in this study. (B) Original CV from ref [24] (Figure 2A), where the grey line in the upper figure A indicates the background current and the black line in the lower figure B indicates the experimental current. (C) CV simulation results: the green dashed line indicates the background current, the light blue dashed line the experimental current, the red dotted line the background-subtracted current, and the dark blue solid line the simulation result. Panel (A) is adapted from Ref [24] and panel (B) is reprinted from Ref [24].

Table S4. Input and fitting parameters used in the CV simulations for Figure S3

Input parameter from the reference [24]			Fitting parameter		
**Parameter**	**Symbol**	**Value**	**Parameter**	**Symbol**	**Value**
Equilibrium potential of NADH	$E^{0'}$	-0.32 V vs SHE (pH 7)	Rate constant at equilibrium potential	k^0	2.0×10^{-4} cm s^{-1}
Electrode area	A	0.09 cm^2	Number of electron transferred in rate determining step	n_a	1
Scan rate	ν	0.025 V s^{-1}	Stretching parameter	γ	2
Concentration of NADH	C_{exp}	1×10^{-7} mol cm^{-3}			
Total Electron transfer number	n	2			

(2) Succinate Oxidation Data 1

In a previous study[25], the mechanism of Succinate oxidation was studied. The Succinate Dehydrogenase (SDH) was added into electrolyte solution containing succinate and buffer. During the CV measurement, SDH adsorbed onto a Pyrolytic Graphite Electrode (PGE) (see Figure S4 (A)). Oxidation current was observed with the equilibrium potential near standard redox potential of succinate oxidation (0.03 V vs SHE at pH 7). Therefore, this data is suitable for studying Succinate oxidation in the complex II. To estimate the overpotential of Succinate oxidation in complex II, the experimental data were extracted from Ref [25] - Fig.1. The original raw data are shown in Figure S4 (B). Then, the simulation curve (Figure S4 (C) – blue line) fitted to the experimental data (see Figure S4 (C) – red dotted line). The input and fitting parameters can be seen in Table S5. Then, using eq.(*2-2*), the j_0 was corrected to physiological concentration (C_{phys}) of succinate which is around 0.5 mM[41] to get j_0.

In Figure S4 (C), the oxidation peak in the simulation fits the experimental data. However, several non-faradaic parts do not fit into the simulation. This may be due to the electric double layer, which was not considered in the simulation. However, it is acceptable since the slope, peak and height of the experimental data and simulation are fit; therefore, we can extract the kinetic parameters.

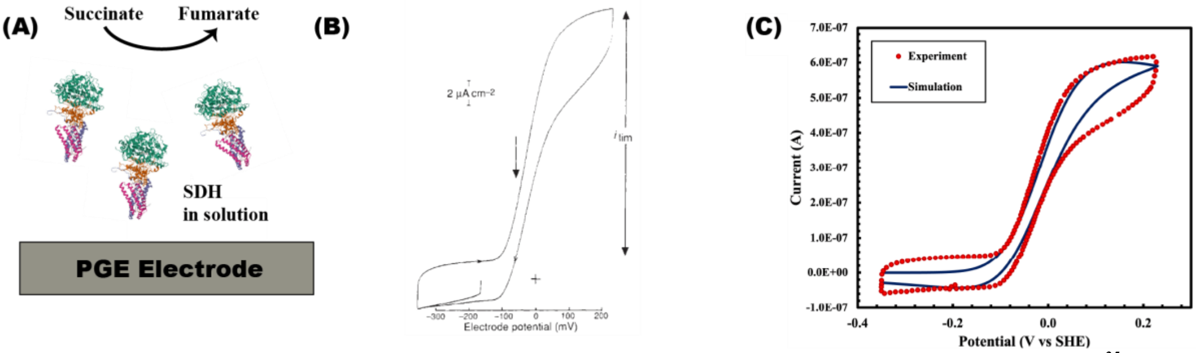

Figure S4. (A) Schematic illustration of the electrochemical system used in this study. (B) Original CV from Ref[25] (Fig. 1). (C) CV simulation results: the red dotted line indicates the background-subtracted current, and the dark blue solid line the simulation result. Panel (B) is reprinted from Ref [24].

Table S5. Input and fitting parameters used in the CV simulations for Figure S4

Input parameter from the reference [25]			Fitting parameter		
**Parameter**	**Symbol**	**Value**	**Parameter**	**Symbol**	**Value**
Equilibrium potential of Succinate	$E^{0'}$	-0.047 V vs SHE (pH 8.3)	Rate constant at Equilibrium potential	k^0	6.0 × 10^{-5} cm s^{-1}
Electrode area	A	0.031 cm^2	Number of electron transferred in rate determining step	n_a	1
Scan rate	ν	0.01 V s^{-1}	Stretching parameter	$\boldsymbol{\gamma}$	6
Concentration of Succinate	C_{exp}	1.0 × 10^{-6} mol cm^{-3}			
Total Electron transfer number	n	2			

(3) Succinate Oxidation Data 2

In a previous study[26], the mechanism of Succinate oxidation was studied. The Succinate Dehydrogenase (SDH) from *Thermus thermophilus* was adsorbed onto a Pyrolytic Graphite Electrode (PGE) (see Figure S5 (A)). There is oxidation current observed with the equilibrium potential near standard redox potential of succinate oxidation (0.03 V vs SHE at pH 7). Therefore, this data is suitable to study Succinate oxidation in the complex II. To estimate the overpotential of Succinate oxidation in complex II, the experimental data were extracted from Ref - Fig.9. The original raw data are shown in Figure S5 (B). Then, the simulation curve (Figure S5 (C) – blue line) fitted to the experimental data (see Figure S5(C) – red dotted line). The input and fitting parameters can be seen in Table S6.

In the reference, the surface coverage by enzyme was not mentioned. Therefore, we calculated it by ourself using the information provided in the reference. It is mentioned the activity of the enzyme (k_{cat}) is 500 min^{-1} and from the CV, the catalytic current (I_{cat}) is around 1.3 × 10^{-6} A. Using surface area of 0.03 cm^2, we got surface coverage as follow:

$$\sigma = \frac{(1.3 \times 10^{-6}\ \mathrm{A})}{(2)(96485)(0.03\ \mathrm{cm}^2)\left(\frac{500}{60\ s}\right)} = 2.7 \times 10^{-11}\ \mathrm{mol\ cm^{-2}}$$

In Figure S5 (C), the oxidation peak in the simulation fits the experimental data. However, several non-faradaic parts do not fit into the simulation. This may be due to the electric double layer, which was not considered in the simulation. However, it is acceptable since the slope, peak and height of the experimental data and simulation are fit; therefore, we can extract the kinetic parameters. Similar to previous data of Succinate oxidation, j_0 was corrected to physiological concentration (C_{phys}) of succinate which is same as previous data.

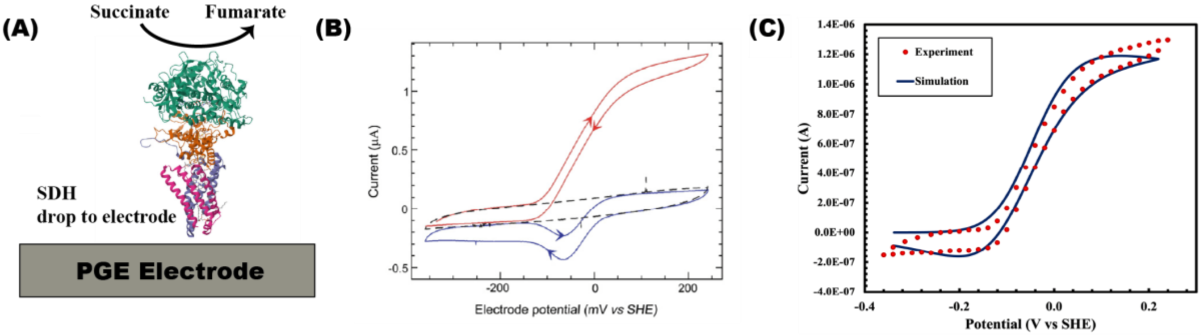

Figure S5. (A) Schematic illustration of the electrochemical system used in this study. (B) Original CV from ref[26] (Fig. 9); the red trace is the succinate CV used for the calculations. (C) CV simulation results: the red dotted line indicates the background-subtracted current, and the dark blue solid line the simulation result Panel (B) reprinted from ref[26]

Table S6. Input and fitting parameters used in the CV simulations for Figure S5

Input parameter from the reference [26]			Fitting parameter		
**Parameter**	**Symbol**	**Value**	**Parameter**	**Symbol**	**Value**
Equilibrium potential of succinate	$E^{0'}$	-0.050 V vs SHE (pH 8.5)	Rate constant at equilibrium potential	k^0	2.0×10^{-4} cm s^{-1}
Electrode area	A	0.030 cm^2	Number of electron transferred in rate determining step	n_a	1
Scan rate	ν	0.005 V s^{-1}	Stretching parameter	γ	7
Concentration of Succinate	C_{exp}	1.0×10^{-6} mol cm^{-3}			
Total Electron transfer number	n	2			

(4) Ubiquinone Reduction and Oxidation Data 1

In a previous study[27], the reaction of quinones in the form of carbazole-HQ immobilized on multiwalled carbon nanotubes-modified glassy carbon (GCE/MWCNT@Car-HQ) was studied (see Figure S6 (A)). This GCE/MWCNT@Car-HQ system is a structural biomimic of Coenzyme 10 or ubiquinone. The original raw data are shown in Figure S6 (B). Although the reaction mechanism is not the same as Q10, the system is stable and shows well defined redox peak around -0.16 V vs Ag/AgCl which is not far from the standard redox potential of Coenzyme 10 (~-0.1V vs Ag/AgCl). Therefore, this data might be useful to estimate the overpotential in the Q10 of the mitochondrial respiratory chain. To estimate the overpotential of the quinone reaction, we extracted the background and experimental data from Ref[27] - Fig.3B (ii) and (iii), respectively. The data were fitted by adjusting k^0 for the peak position and C for the peak height. The input and fitting parameter can be seen in Table S7. The redox potential shifted about 0.015V in the simulation, and we considered this acceptable because the shifted is so small.

In the Figure S6 (C), the oxidation and reduction peaks of the simulation fitted those of the experiment. However, some areas near the oxidation peak do not fit the simulation. This may be due to the electric double layer or IR drop, which was not covered by the simulation. However, this was acceptable because the peak and height around the redox reaction simulation were fitted, and the kinetic parameters could be extracted. Then, using eq.(2-2) the $j_{0\ exp}$ was corrected to physiological concentration (C_{phys}) of CoQ_{10} in inner membrane mitochondria which is around 5 mM[42] to get j_0.

Since the redox potential of this system is shifting about 0.015 V , therefore, for the ubiquinone reaction, we shift the obtained potential by 0.015V. For example, at the Γ_e = 4 e$^-$ site^{-1} s^{-1}, we estimated η= 0.16 V for Car-HQ system, the corresponding ubiquinone overpotential becomes :

$$|\eta_{UQ}| = |\eta_{Car-HQ}| - 0.015\ V \approx 0.15V$$

Thus, using ubiquinone physiological relevant redox potential, reduces the overpotential magnitude from 0.16 V to 0.085 V. We also evaluated the impact on the exchange current density using Tafel relation

$|\eta| = b\ \log_{10}\left(\frac{|j|}{|j_0|}\right)$, with α = 0.5, and b= 0.12 V/dec

For the same current j :

$$\log_{10} j_{0,Car} = \log_{10} j - \frac{|\eta_{Car}|}{b} = \log_{10} j - \frac{0.16}{b}$$

$$\log_{10} j_{0,UQ} = \log_{10} j - \frac{|\eta_{UQ}|}{b} = \log_{10} j - \frac{0.15}{b}$$

Therefore,

$$\frac{j_{0,UQ}}{j_{0,Car-HQ}} = 10^{\frac{|\eta_{Car-HQ}|-|\eta_{UQ}|}{b}} = 10^{\frac{0.16-0.15}{0.12}} = 10^{0.08} \approx 1.2$$

So, using Tafel relation, the exchange current density (j_0) referenced to ubiquinone is larger by a factor of 1.2 compared to Car-HQ value. Because this is a uniform shift in η and j_0, it does not affect catalytic kinetics. Sensitivity analysis of j_0 was performed to show that qualitative behaviour and all key conclusion of the model remain unchanged after this shift.

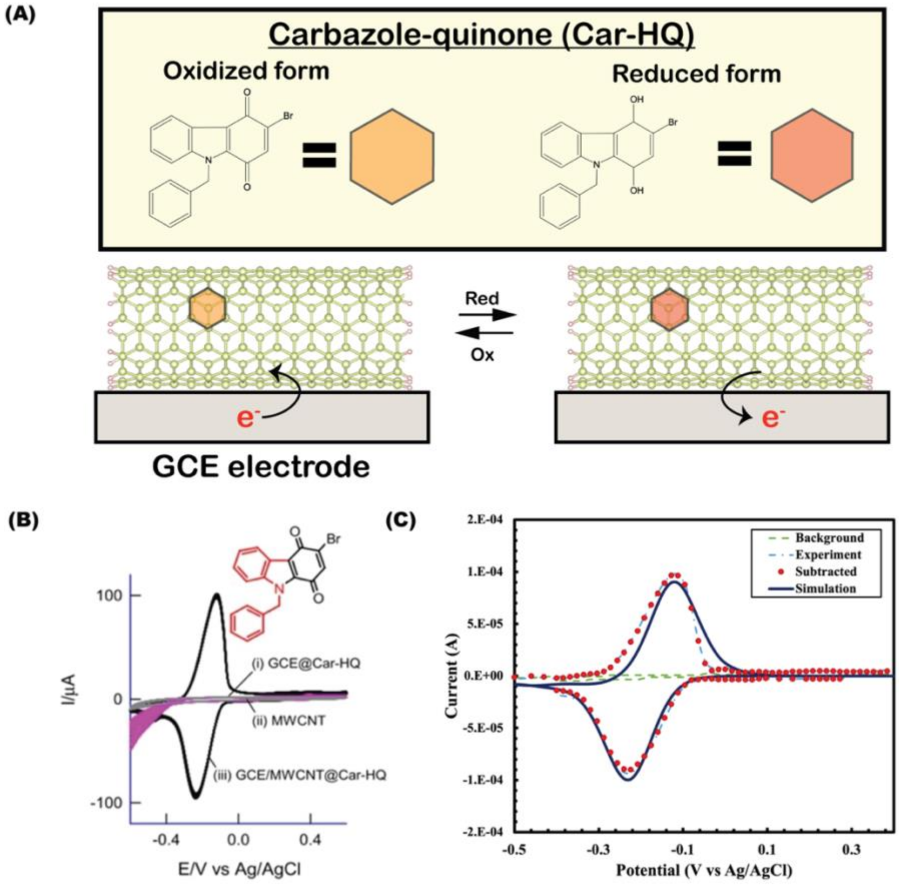

Figure S6. (A) Schematic illustration of the electrochemical system used in this study. (B) Original CV from ref[26] (Figure 3b); grey line (ii), represents background current and black line (iii) represents experimental current. (C) CV simulation results: the green dashed line indicates the background current, the light blue dashed line the experimental current, the red dotted line the background-subtracted current, and the dark blue solid line the simulation result. Panel (B) reprinted from the ref[27].

Table S7. Input and fitting parameters used in the CV simulations for Figure S6

Input parameter from the reference[27]			Fitting parameter		
**Parameter**	**Symbol**	**Value**	**Parameter**	**Symbol**	**Value**
Equilibrium potential of Car-HQ	E^{0}’	-0.175 V vs Ag/AgCl (pH 7)	Rate constant at equilibrium potential	k^0	1.5×10^{-3} cm s^{-1}
Electrode area	A	0.0707 cm^2	Number of electron transferred in rate determining step	n_a	1
Scan rate	ν	0.05 V s^{-1}	Stretching parameter	γ	0.3
Concentration of Car-HQ	C_{exp}	5.0×10^{-6} mol cm^{-3}	Shifting parameter to ubiquinone sistem	ΔE^{0}’	0.015 V

Total Electron transfer number	n	3			

(5) Ubiquinone Reduction and Oxidation Data 2

In a previous study[28], a biomimetic system of ubiquinone (UQ) reduction was studied using coenzyme Q10 immobilized in functionalized multiwalled carbon nanotubes (GC/f-MWCNT@Q10) (see. Figure S7 (A)). This system biomimic the Q10 system. Although the electron transfer mechanism here involves two electrons which is not the same as physiological Q10, at pH 13 the peak-to-peak separation was small and close to the physiological condition of Q10. Therefore, this data might be useful to estimate overpotential in Ubiquinone. To estimate the overpotential in this reaction, experimental data were extracted from Ref [28]- Fig.8 (A) at pH 13. The original raw data is shown in Figure S7 (B) . However, this reference did not provide background data; therefore, the capacitive current was used as background data (see Figure S7 (C) – green line), which had a rectangular shape[43]. The input and fitting parameters can be seen in Table S8. The oxidation peak was slightly lower than the reduction peak, which means that some part of ubiquinone was removed from the reaction, therefore we added second-order homogeneous rate constant (k_{R2}) for around 5000 cm^3 mol^{-1} s^{-1} to fit the simulation peak to the experiment.
In Figure S7 (C), the oxidation and reduction peaks of the simulation fitted those of the experiment. Some areas after the peak are not fitted. This may be due to some IR drop or other experimental factors which was not covered by the simulation. However, this was acceptable because the peak and height of the redox reaction almost fit the experiment. Similar to previous data of Ubiquinone reduction oxidation, j_0 was corrected to physiological concentration (C_{phys}) of CoQ_{10} in Inner Membrane Mitochondria (IMM) which is same as previous data.

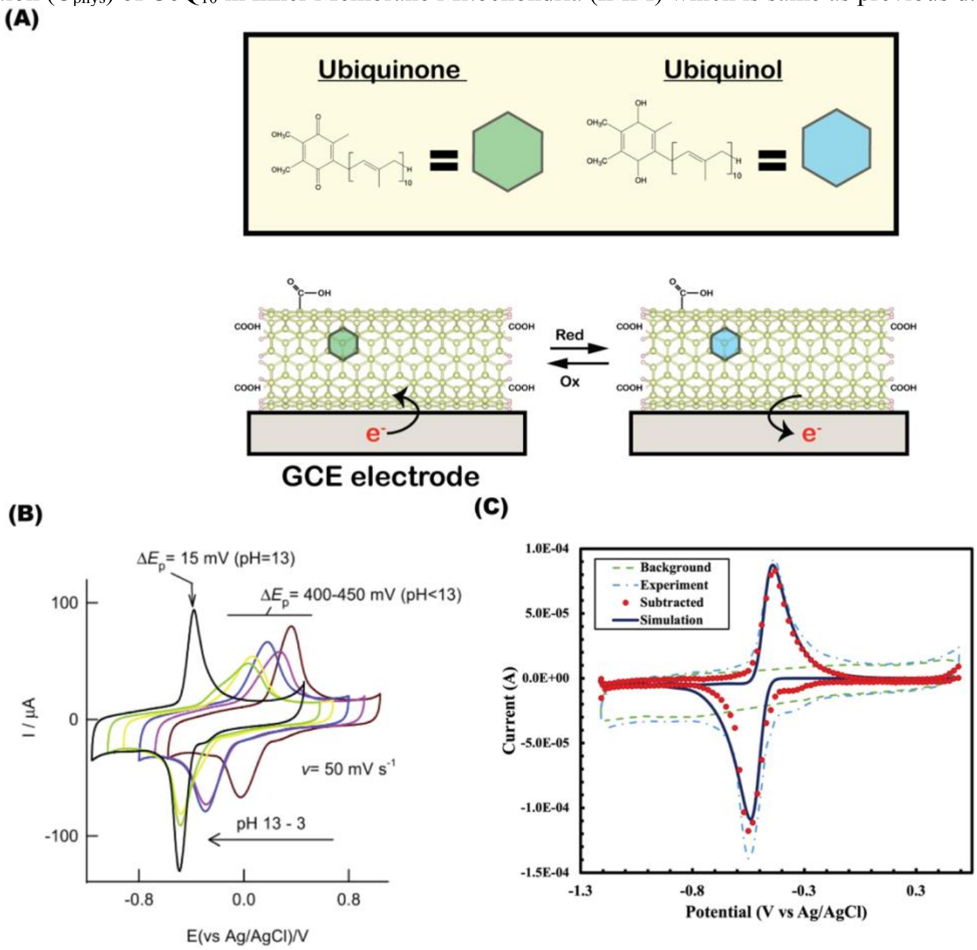

Figure S7. (A) Schematic illustration of the electrochemical system used in this study.(B) Original CV from ref[28] (Figure 8A); the pH 13 trace represents the experimental data used in this work. (C) CV simulation results: the green dashed line indicates the background current, the light blue dashed line the experimental current, the red dotted line the background-subtracted current, and the dark blue solid line the simulation result. Panel (B) reprinted from the Ref[28].

Table S8. Input and fitting parameters used in the CV simulations for Figure S7

Input parameter from the reference [28]			Fitting parameter		
**Parameter**	**Symbol**	**Value**	**Parameter**	**Symbol**	**Value**
Equilibrium potential of Q10	$E^{0'}$	-0.44 V vs Ag/AgCl (pH 13)	Rate constant at equilibrium potential	k^0	1.5×10^{-2} cm s^{-1}
Electrode area	A	0.0707 cm^2	Number of electron transferred in rate determining step	n_a	2
Scan rate	ν	0.05 V s^{-1}	Stretching parameter	$\boldsymbol{\gamma}$	0.6
Concentration of Q10	C_{exp}	5.0×10^{-7} mol cm^{-3}	Second order Homogeneous rate constant	$k_{R,2}$	5000 cm^3 mol^{-1} s^{-1}
Total Electron transfer number	n	2			

(6) Ubiquinone Reduction and Oxidation Data 3

In a previous study[29], the redox reaction of ubiquinone was studied by using carboxylic acid-functionalized hydroquinone (H_2Q-COOH) immobilized on multiwalled carbon nanotube modified glassy carbon electrode (GCE/MWCNT). The system used in the experiment shown in the see Figure S8 (A). A well-defined redox peak similar to that of the ubiquinone system was observed and the reaction mechanism is similar to ubiquinone which involved one electron transfer mechanism. Therefore, this data can be used to estimate the overpotential in ubiquinone. To calculate the overpotential, experimental data were extracted from Reference[29] - Fig.2 (A) at a scan rate of 10 mV s^{-1}. The original raw data shown in Figure S8 (B). Here, only peak A1/C1 was considered which shows the reaction of Hydroquinone (H_2Q) and semiquinone (·QH) one electron transfer reactions. The CV in the supplementary data of Ref [29] – Fig.S8 was used as the background data by slightly extending the capacitive current (see Figure S8 (C) – green lines).
The input and fitting parameter can be seen in Table S9. In Figure S8 (C), the oxidation and reduction peaks of the A1/C1 in simulation fitted those of the experiment. However, some areas did not fit the simulation results. This may be due to the IR drop or other experimental conditions which were not covered by the simulation. However, this was acceptable because the peak and height of the reaction simulation almost fit those of peak A1/C1; therefore, the kinetic parameters could be extracted. Similar to previous data of Ubiquinone reduction oxidation, $j_{0\ \mathrm{exp}}$ was corrected to physiological concentration (C_{phys}) of CoQ_{10} in IMM which is same as previous data.

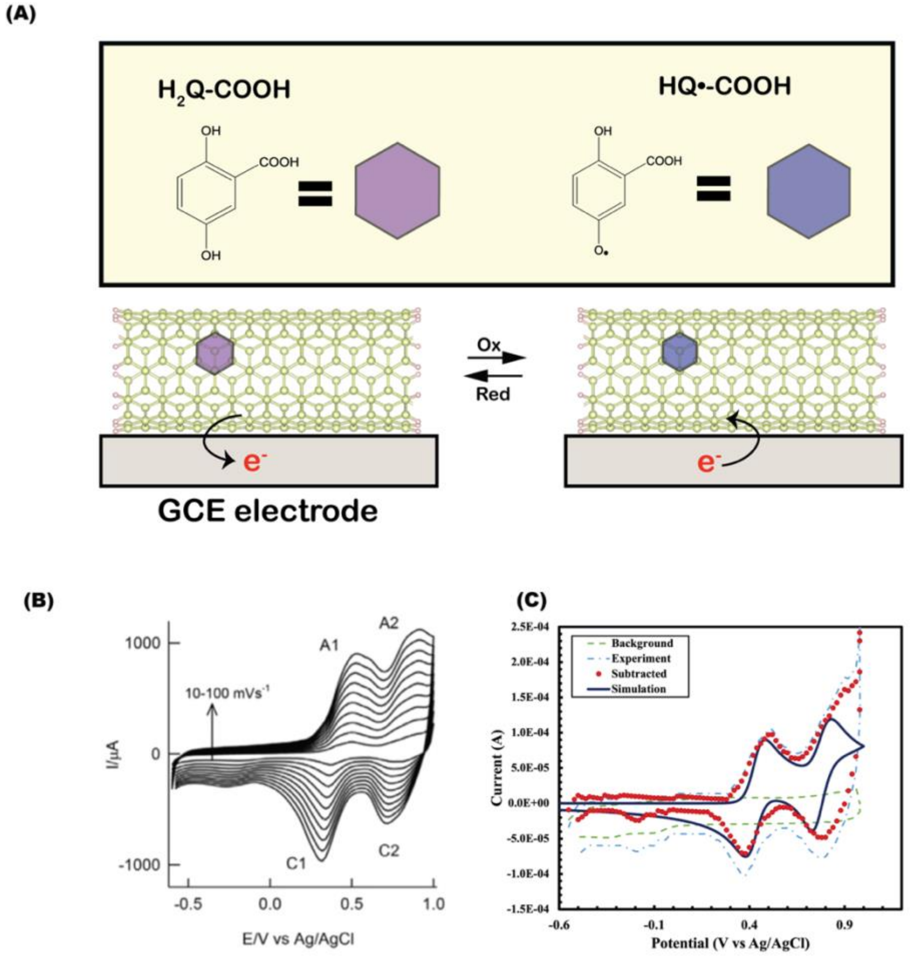

Figure S8. (A) Schematic illustration of the electrochemical system used in this study. (B) Original CV from Ref[29] (Figure 2A); the scan at 10 mVs^{-1} represents the experimental data used in this study. (C) CV simulation results: the green dashed line indicates the background current, the light blue dashed line the experimental current, the red dotted line the background-subtracted current, and the dark blue solid line the simulation result. Panel (B) reprinted from the Ref[29].

Table S9. Input and fitting parameters used in the CV simulations for Figure S8

Input parameter from the reference[29]			Fitting parameter		
**Parameter**	**Symbol**	**Value**	**Parameter**	**Symbol**	**Value**
Equilibrium potential of H_2Q	$E^{0'}$	0.43 V vs Ag/AgCl (pH 2)	Rate constant at equilibrium redox potential	k^0	1 cm s^{-1}
Electrode area	A	0.0707 cm^2	Number of electron transferred in rate determining step	n_a	1
Scan rate	ν	0.01 V s^{-1}			
Concentration of H_2Q-COOH	C_{exp}	1.0×10^{-7} mol cm^{-3}			
Total Electron transfer number	n	1			

(7) Cytochrome c Reduction and Oxidation Data 1

In a previous study[44], the redox reaction of cytochrome c was studied a solution of cytochrome c and gold electrode modified with a Metal Schiff Base (M-SB). The system used in the experiment is shown in the see Figure S9(A). This system was similar to the active site of cytochrome c in the respiratory system. It exhibited a good electrochemical response similar to that of cytochrome c, under physiological conditions. Therefore, this data

might be suitable to study cytochrome c in the mitochondrial respiratory chain. To calculate the overpotential, the background and experimental data were extracted from Supplementary Fig. S2 in Reference [44]. Figure S9 (B) presents the original raw data CV with and without cytochrome c used as experimental and background data, respectively.

The input and fitting parameters can be seen in Table S10. In the Figure S9 (C), the peak in the simulation and the experiment curves were successfully adjusted. However, some areas did not fit the simulation results. This may be due to the electric double layer or IR drop, which was not covered by the simulation. Then, using eq.(2-2) the $j_{0\ exp}$ was corrected to physiological concentration (C_{phys}) of Cytochrome c which is around 0.5 mM[45] to get j_0.

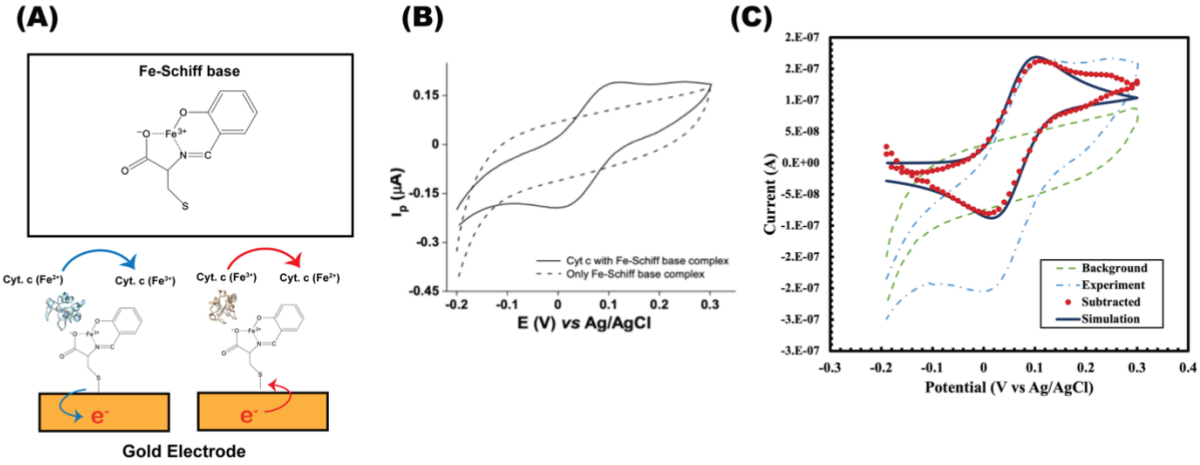

Figure S9. (A) Schematic illustration of the electrochemical system used in this study. (B) Original CV from Ref[30] (Figure S2); the dashed line represents the background current and the solid line represents the experimental current. (C) CV simulation results: the green dashed line indicates the background current, the light blue dashed line the experimental current, the red dotted line the background-subtracted current, and the dark blue solid line the simulation result. Panel (A) is adapted from ref [30], and panel (B) is reprinted from Ref [30].

Table S10. Input and fitting parameters used in the CV simulations for Figure S7

Input parameter from the reference [44]			Fitting parameter		
**Parameter**	**Symbol**	**Value**	**Parameter**	**Symbol**	**Value**
Equilibrium potential of Cytochrome c	$E^{0'}$	0.06 V vs Ag/AgCl (pH 7)	Rate constant at equilibrium potential	k^0	5.0×10^{-2} cm s^{-1}
Electrode area	A	0.0314 cm^2	Number of electron transferred in rate determining step	n_a	1
Scan rate	ν	0.03 V s^{-1}			
Concentration of Cytochrome c	C_{exp}	5.0×10^{-8} mol cm^{-3}			
Total Electron transfer number	n	1			

(8) Cytochrome c Reduction and Oxidation Data 2

In a previous study[31], the redox reaction of cytochrome c was studied using cardiolipin deposited on a glassy carbon electrode (GCE). This reaction system mimics the interface of the inner mitochondrial membrane and promotes the reaction of cytochrome c. Therefore, this data might be useful to study cytochrome c reaction in the mitochondrial respiratory system. The system used in the experiment is shown in the Figure S10 (A). To estimate the overpotential of the cytochrome c reaction, the background and experimental data were extracted from the Reference [31] - Fig.4A black and blue lines, respectively. Figure S10 (B) shows the original raw data.
The input and fitting parameters can be seen in Table S11. In the Figure S10 (C), the peak position of the simulation and experiment data were successfully adjusted. The oxidation peak was lower than the reduction peak,

which means that some part of cytochrome c was removed from the reaction, therefore we added second order homogeneous rate constant (k_{R2}) for around 1.0×10^5 cm^3 mol^{-1} s^{-1} to fit the simulation peak to the experiment. In the reference, 7 μg cm^{-2} of cardiolipin was deposited in the electrode. Therefore, using the molecular weight of cardiolipin ($C_{81}H_{148}O_{17}P_2Na_2$), which was 1501.95 mol g^{-1} , its coverage on the electrode was estimated as follows

$$\sigma = \frac{7 \times 10^{-6} \text{ g cm}^{-2}}{1501.95 \text{ g mol}^{-1}} = 4.6 \times 10^{-9} \text{ mol cm}^{-2}$$

Assuming that all cytochrome c is attached to the cardiolipin during the reaction, we can say that σ of cardiolipin is the same with coverage of cytochrome c. Similar to previous data of Cytochrome c reduction oxidation, j_0 was corrected to physiological concentration (C_{phys}) of Cytochrome c which is same as previous data.

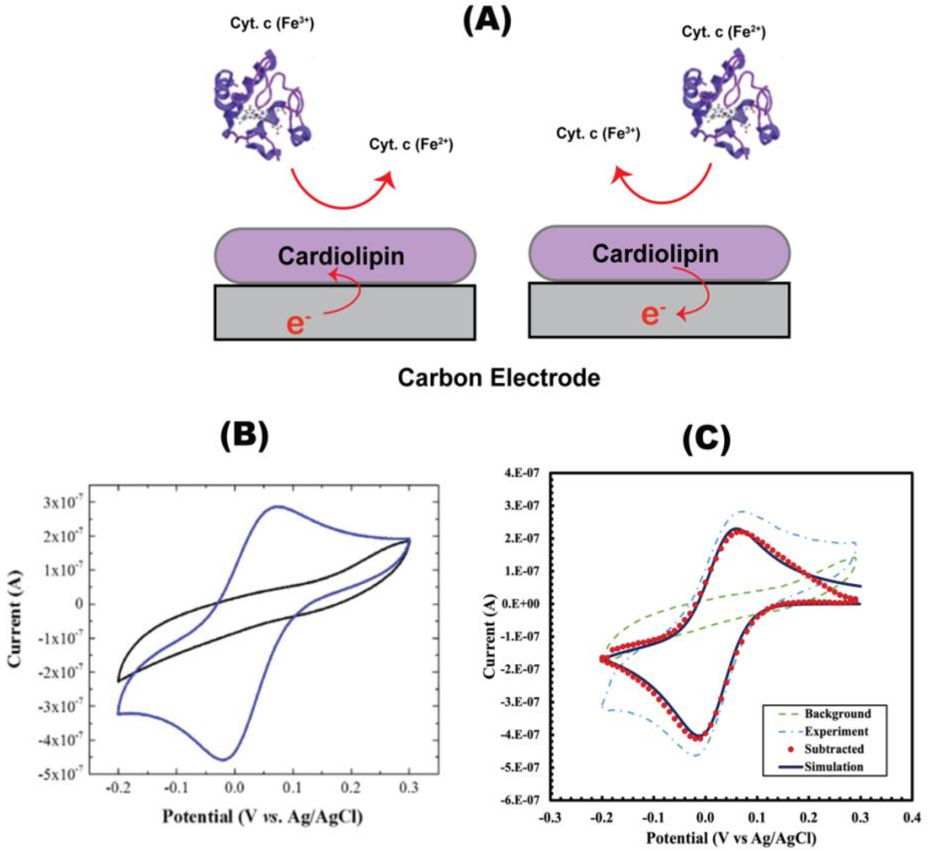

Figure S10. (A) Schematic illustration of the electrochemical system used in this study. (B) Original CV from Ref[31] (Figure 4A); the black line represents the background current (without cytochrome c) and the blue line represents the experimental current (with cytochrome c). (C) CV simulation results: the green dashed line indicates the background current, the light blue dashed line the experimental current, the red dotted line the background-subtracted current, and the dark blue solid line the simulation result. Panel (A) is adapted from ref[30], and panel (B) is reprinted from Ref[31].

Table S11. Input and fitting parameters used in the CV simulations for Figure S10

Input parameter from the reference[31]			Fitting parameter		
**Parameter**	**Symbol**	**Value**	**Parameter**	**Symbol**	**Value**
Equilibrium potential of Cytochrome c	$E^{0'}$	0.023 V vs Ag/AgCl (pH 7)	Rate constant at equilibrium potential	k^0	1.0 cm s^{-1}
Electrode area	A	0.071 cm^2	Number of electron transferred in rate determining step	n_a	1
Scan rate	ν	0.02 V s^{-1}	Second order Homogeneous rate constant	$k_{R,2}$	1.0×10^5 cm^3 mol^{-1} s^{-1}

Concentration of Cytochrome c	C_{exp}	1.5×10^{-7} mol cm^{-3}			
Total Electron transfer number	n	1			

S2-2 Analysis of Linear Sweep Voltammetry (LSV) data to obtain exchange current density (j_0)

The LSV data were analyzed using the Tafel slopes. First, screen-captured the graphs from the references listed in Table S3. Then, WebPlotDigitizer [39] was used to extract raw data from the graph in the form of .csv file. Next, constructed a Tafel plot (log $|j|$ vs overpotential) using the raw data and calculated the exchange current density ($j_{0\ \mathrm{exp}}$). Then, using eq.(*2-2*), we converted the $j_{0\ \mathrm{exp}}$ to j_0. The exchange electron transfer frequency (Γ_{e0}) was calculated using eq (2-3). Finally, using all these parameters, the overpotential for a specific Γ_e was calculated using modified the Butler-Volmer equation in eq (4).

$$\Gamma_e = \Gamma_{e0}\left(e^{\frac{2.3}{\mathrm{slope}}\eta} - e^{-\frac{2.3}{\mathrm{slope}}\eta}\right) \tag{4}$$

For all of the ORR data, $j_{0\ \mathrm{exp}}$ was corrected from concentration of saturated oxygen in the water which is 0.26 mM to oxygen concentration in mitochondria (C_{phys}) which is around 0.01 mM to get j_0 using eq.(*2-2*) .[46]

<u>Fitting Result:</u>

(1) ORR data 1

In a previous study[32], the ORR was studied using the synthetic heme-Cu (FeCu) of complex IV. The system is shown in Figure S11 (A) where the synthetic active site was absorbed on an edge plane graphite (EPG) electrode at pH 7 in an aqueous condition. It shows $4e^-$ oxygen reduction reaction to H_2O just like Complex IV and high catalytic activity (4.49×10^6 $M^{-1}s^{-1}$) comparable to native complex IV (~10^6 – 10^7 $M^{-1}s^{-1}$). To calculate the overpotential, we extracted the experimental data from Ref.[32] - Fig. 4C at 500 rpm. The Figure S11 (B) shows the original raw data. The Tafel plot is shown in Figure S11 (C) with $E^{0'}$ = 0.816 V vs SHE. The input and fitting parameters used in the tafel plot are shown in Table S12. Using this information, the overpotential for a specific Γ_e was estimated using abovementioned method.

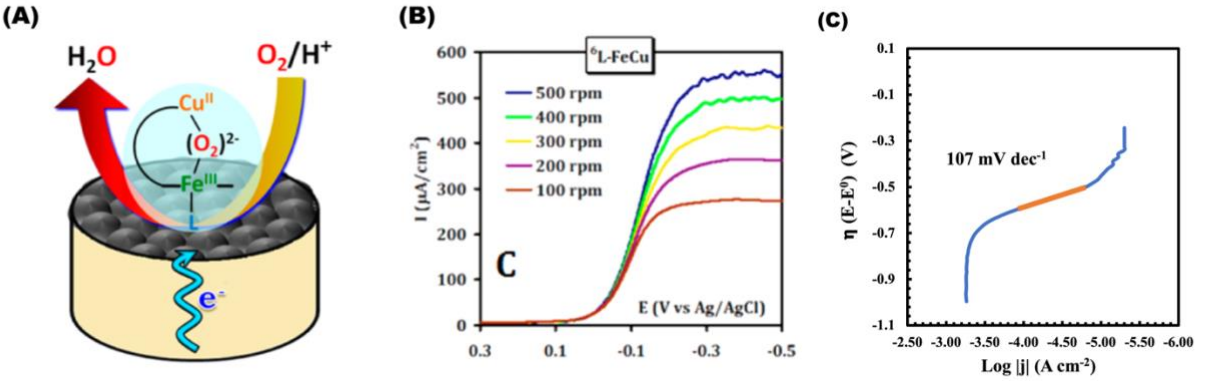

Figure S11. (A) Schematic illustration of the electrochemical system used in this study. (B) Original LSV from Ref [32] (Figure 4C); the data at 500 rpm were used for our calculation. (C) Replotted Tafel plot derived from (B) using $E^{0'}$ = 0.816 V. The orange line indicates the Tafel slope used for the overpotential calculation. Panels (A) and (B) are reprinted from ref [32]

Table S 12. Input and fitted parameters used in the Tafel analysis for Figure S11

Input parameter from the reference[32]			Fitting parameter		
Parameter	Symbol	Value	Parameter	Symbol	Value
Standard redox potential of ORR	E^{0}'	0.816 V vs SHE (pH 7)	Slope	2.3RT/αzF	107 mV dec^{-1}
			Exchange current density	$j_{0\ exp}$	3.1 × 10^{-10} A cm^{-2}

(2) ORR data 2

In a previous study[33], the ORR was studied using a biosynthetic model of complex IV. The system used for the experiment is shown in the Figure S12 (A) where the active sites from the myoglobin mutants were used as the ORR active sites because they have similar active sites as complex IV. The result shows 4e- ORR process and it shows high ORR rates (1.98 × 10^{7} M^{-1}s^{-1}) comparable to native complex IV (~10^{6} – 10^{7} M^{-1}s^{-1}). To calculate the overpotential, the experimental data were extracted from the LSV plot in the Ref [33] Fig. 4 C. The original raw data shown in Figure S12 (B). The Tafel plot is shown in Figure S12 (C) with E^{0}' = 0.816 V vs SHE. The input and fitting parameters used in the tafel plot are shown in Table S13. Using this information, the overpotential for a specific Γ_e was estimated using the abovementioned method.

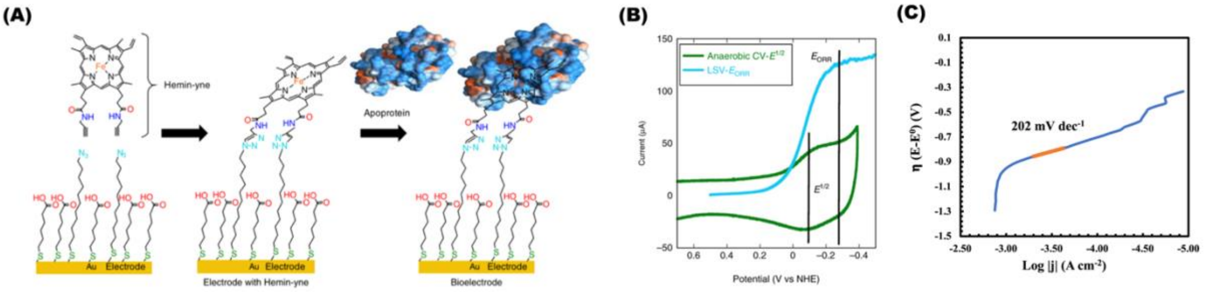

Figure S12. (A) Schematic illustration of the electrochemical system used in this study. (B) Original LSV data from Ref[33] (Figure 4C), used for the overpotential estimation. (C) Replotted Tafel plot derived from (B) using E^{0}' = 0.816 V. The orange line indicates the Tafel slope used for the overpotential calculation. Panels (A) and (B) are reprinted from the Ref [33].

Table S13. Input and fitted parameters used in the Tafel analysis for Figure S12

**Input parameter from the reference[33]**			**Fitting parameter**		
**Parameter**	**Symbol**	**Value**	**Parameter**	**Symbol**	**Value**
Standard redox potential of ORR	E^{0}'	0.816 V vs SHE (pH 7)	Slope	2.3RT/αzF	202 mV dec^{-1}
			Exchange current density	$j_{0\ exp}$	2.8 × 10^{-8} A cm^{-2}

(3) ORR data 3

In a previous study[34], ORR was studied using synthetic mononuclear iron porphyrins that mimicked the active sites of complex IV. The system use in the experiment shown in the. Figure S13 (A). It exhibits a high catalytic activity (1.35 × 10^{7} M^{-1}s^{-1}) comparable to native complex IV (~10^{6} – 10^{7} M^{-1}s^{-1}) and good 4e^{-} oxygen reduction reaction selectivity. To calculate the overpotential, the experimental data were extracted from the LSV plot in the Ref [34] Fig. 3E at 500 rpm. The original raw data is shown in Figure S13 (B). The Tafel plot is shown in Figure S13 (C) with E^{0}' = 0.816 V vs SHE. The input and fitting parameters used in the tafel plot are shown in Table S14. Using this information, the overpotential for a specific Γ_e was estimated using the abovementioned method.

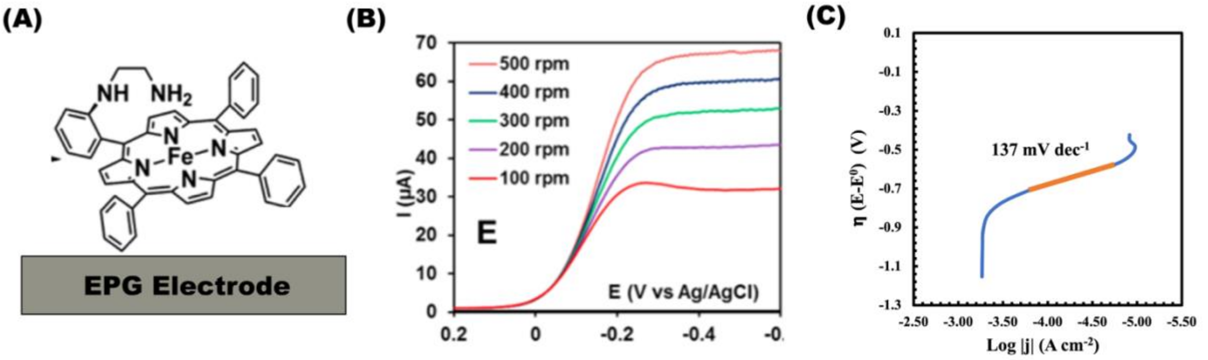

Figure S 13. (A) Schematic illustration of the electrochemical system used in this study. (B) Original LSV from Ref [34] (Figure 3E); the data at 500 rpm were used for our calculation. (C) Replotted Tafel plot derived from (B) using E^{0}' = 0.816 V. The orange line indicates the Tafel slope used for the overpotential calculation. Panel (A) is adapted from Ref[34] and panel (B) is reprinted from the Ref [34].

Table S14. Input and fitted parameters used in the Tafel analysis for Figure S13

Input parameter from the reference[34]			Fitting parameter		
**Parameter**	**Symbol**	**Value**	**Parameter**	**Symbol**	**Value**
Standard redox potential of ORR	E^{0}'	0.816 V vs SHE (pH 7)	Slope	2.3RT/αzF	137 mV dec^{-1}
			Exchange current density	$j_{0\ exp}$	1.1 × 10^{-9} A cm^{-2}

(4) ORR data 4

In a previous study[35], the ORR was studied using Fe-Cu dual atomic sites embedded in 3D porous N-doped carbon (FeCu-DA/NC), which mimicked the active site of complex IV. The system used in the experiment is shown in the Figure S14(A) and the experiment was conduct in acid condition with the solution of 0.5 M H_2SO_4. It exhibits good selectivity of 4e- oxygen reduction reaction (ORR) and ORR mechanism similar to complex IV where the Fe is mainly contributed in the ORR. To calculate the overpotential, the experimental data were extracted from the LSV plot in the Ref [35] - Fig. 3D. The original raw data is shown in the Figure S14 (B). In the reference, the coverage of the active site was not mentioned. Therefore, we estimated by ourself as follows:

It was mentioned that the amount of Fe and Cu in FeCu-DA/NC were 1.33 wt% and 0.84 wt%, respectively. Therefore, the total amount of metal in the catalyst was 2.17 wt%. The catalyst loading used in the reference was 0.5 mg cm^{-2}. Then, using the information of FeCu molecular weight, 119.391 g mol^{-1}, σ was estimated

$$\sigma = \frac{(5 \times 10^{-4}\ \mathrm{g\ cm^{-2}})(0.0217)}{119.391\ \mathrm{g\ mol^{-1}}} = 9.0 \times 10^{-8}\ \mathrm{mol\ cm^{-2}}$$

The Tafel plot is shown in Figure S14 (C) with E^{0}' = 1.23 V vs RHE. The input and fitting parameters used in the Tafel plot are shown in Table S13. Using this information, the overpotential for a specific Γ_e was estimated using the abovementioned method.

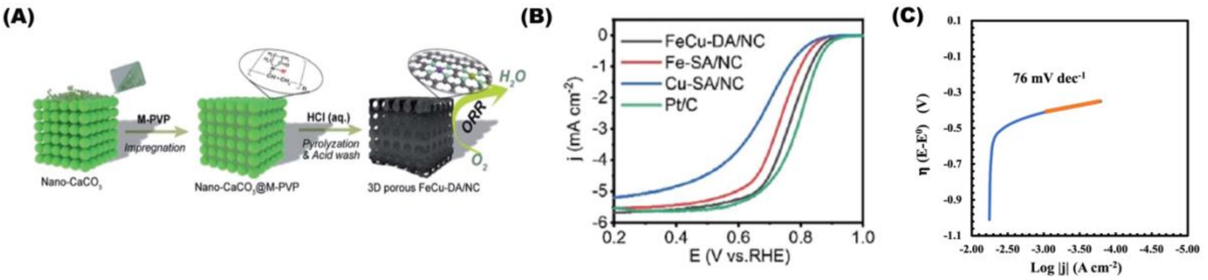

Figure S14. (A) Schematic illustration of the electrochemical system used in this study. (B) Original LSV from Ref[35] (Figure 3D); the FeCu-DA/NC data (black line) were used for our calculation. (C) Replotted Tafel plot derived from (B) using E^{0}' =

1.23 V. The orange line indicates the Tafel slope used for the overpotential calculation. Panels (A) and (B) are reprinted from Ref[35].

Table S15. Input and fitted parameters used in the Tafel analysis for Figure S14

Input parameter from the reference [35]			Fitting parameter		
**Parameter**	**Symbol**	**Value**	**Parameter**	**Symbol**	**Value**
Standard redox potential of ORR	$E^{0'}$	1.23 V vs RHE	Slope	2.3RT/αzF	76 mV dec^{-1}
			Exchange current density	$j_{0\ exp}$	4.3 × 10^{-9} A cm^{-2}

(5) ORR data 5

In a previous study[35], the ORR was studied using Fe-Cu dual atomic sites embedded in 3D porous N-doped carbon (FeCu-DA/NC), which mimicked the active site of complex IV. The system used in the experiment is the same with previous data in the Figure S14 (A). However, a base condition with 0.1 M KOH solution was used here. The experiment shows good selectivity of 4e- oxygen reduction reaction (ORR) and lower overpotential than Pt/C catalyst which was expected in the complex IV. To calculate the overpotential, the experimental data were extracted from the LSV plot in the Ref [35] - Fig. 3A. The original raw data is shown in Figure S15 (A). In the reference, the coverage of the active site was not mentioned. However, these data used the same amount as the previous data, and the only difference was the electrolyte, which used alkaline conditions for these data, and acidic for the previous one. The Tafel plot is shown in Figure S15 (B) with $E^{0'}$ = 1.23 V vs RHE. The input and fitting parameters used in the Tafel plot are shown in Table S16. Using this information, the overpotential for a specific Γ_e was estimated using the abovementioned method.

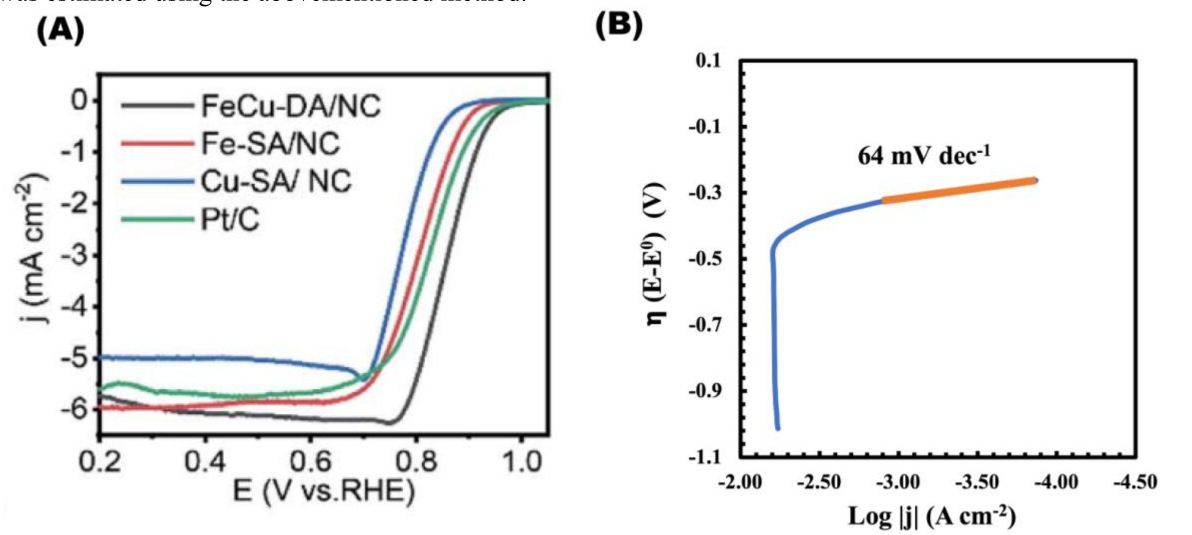

Figure S15. (A) Original LSV from Ref[35] (Figure 3A); the FeCu-DA/NC data (black line) were used for our calculation. (B) Replotted Tafel plot derived from (B) using $E^{0'}$ = 1.23 V. The orange line indicates the Tafel slope used for the overpotential calculation. Panel (A) is reprinted from ref[35].

Table S16. Input and fitted parameters used in the Tafel analysis for Figure S15

Input parameter from the reference[35]			Fitting parameter		
**Parameter**	**Symbol**	**Value**	**Parameter**	**Symbol**	**Value**
Standard redox potential of ORR	$E^{0'}$	1.23 V vs RHE (pH 7)	Slope	2.3RT/αzF	64 mV dec^{-1}
			Exchange current density	$j_{0\ exp}$	1.02 × 10^{-8} A cm^{-2}

The LSV data for the ORR in data 4 and 5 were obtained under acidic and alkaline conditions, respectively. Since the data are reported with respect to the reversible hydrogen electrode (RHE), which inherently includes pH dependence[47], the electrode potential can be directly compared to physiological pH (7.4).

For the exchange current density (j_0), given that pH (7.4) lies between the acidic and alkaline limits, we used the j_0 values from ORR data 4 (acidic) and 5 (alkaline) as lower and upper bounds for the expected kinetic range, with overpotential falling within this range.
To validate this approach, we performed a sensitivity analysis by estimating j_0 and the Tafel slope at pH 7.4 using linear approximation from ORR data 4 and 5 , and assessed its impact on the overpotential (see Figure S16). While we acknowledge that the ORR mechanism at pH 7.4 may differ from those at acidic and alkaline condition, we believe that the data from these extreme conditions provide a reasonable estimation. Future work involving direct measurement at pH 7.4 would provide a more accurate characterization of ORR kinetics in physiological environments.

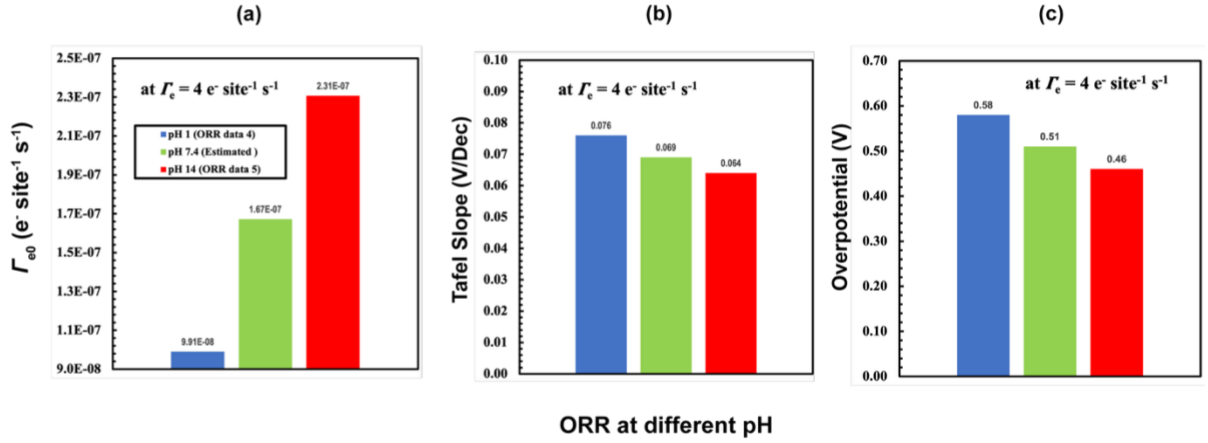

Figure S16. Sensitivity analysis based on ORR data from ref[35] . Blue bars correspond to acidic-condition data (ORR data 4), red bars to alkaline-condition data (ORR data 5), and green bars to values estimated at physiological pH 7.4. The green values in (a) and (b) were obtained by linear interpolation between the acidic and alkaline data. (a) Comparison of exchange electron transfer frequency (Γ_{e0}) at the three pH conditions. (b) Comparison of Tafel slope at three pH conditions. (c) Comparison of overpotential at the three pH conditions, calculated using the Butler-Volmer equation.

(6) ORR data 6

In a previous study[36], the ORR was studied using the bionic design of cytochrome c oxidase (CcO) -like FeN_5 single atom nano enzymes (FeN_5 SAs). The system used in the experiment is shown in the Figure S17 (A). The FeN_5 SAs structures mimic the heme structure of ORR active site in CcO. Cytochrome c was used to provide electrons for ORR and It shows affinity around $K_m = 4.2 \times 10^{-5}$ M to cytochrome c, comparable to those natural CcO ($K_m = 10^{-5} - 10^{-8}$ M). The ORR activity shows selectivity of 4e- ORR process and lower overpotential compared to Pt/C just as expected from the CcO activity. To calculate the overpotential, the experimental data were extracted from the LSV plot in the Ref[36] - Fig. S4 b. The original raw data is shown in Figure S17 (B). In the reference, the coverage of the active site was not mentioned. Therefore, we estimated it as follow:

It was mentioned that the amount of Fe in the FeN_5 SAs was 1.2 wt%. The catalyst loading in the reference was 200 μg cm^{-2}. Then, using the information of Fe molecular weight, 55.845 g mol^{-1}, σ was estimated

$$\sigma = \frac{(2 \times 10^{-4}\ \text{g cm}^{-2})(0.012)}{55.845\ \text{g mol}^{-1}} = 4.3 \times 10^{-8}\ \text{mol cm}^{-2}$$

The Tafel plot is shown in Figure S16 (C) with $E^{0'}$ = 1.23 V vs RHE. The input and fitting parameters used in the Tafel plot are shown in Table S17. Using this information, the overpotential for a specific Γ_e was estimated using the abovementioned method.

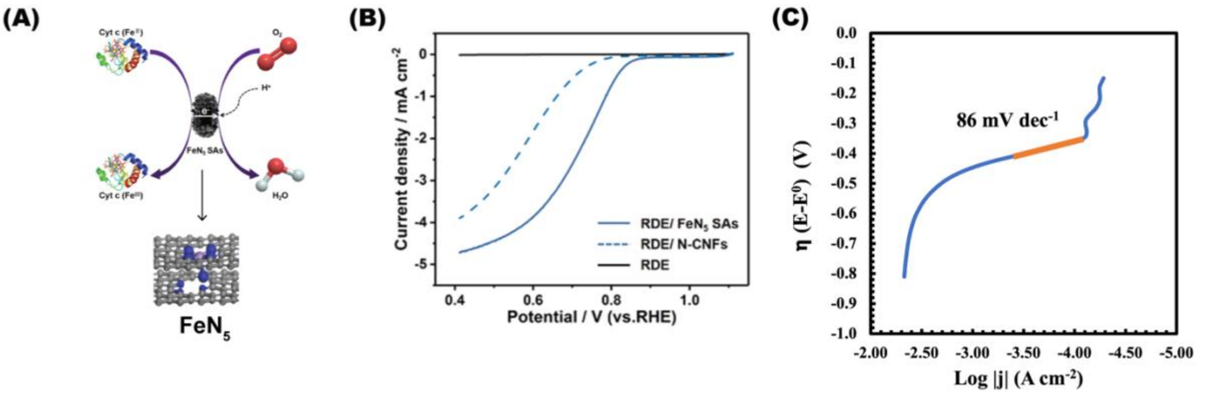

Figure S17. (A) Schematic illustration of the electrochemical system used in this study. (B) Original LSV from Ref[36] (Figure S4); the FeN_5 SAs data were used for our calculation. (C) Replotted Tafel plot derived from (B) using $E^{0'}$ = 1.23 V. The orange line indicates the Tafel slope used for the overpotential calculation. Panels (A) and (B) was reprinted from the ref[36].

Table S17. Input and fitted parameters used in the Tafel analysis for Figure S17

**Input parameter from the reference**[35]			**Fitting parameter**		
**Parameter**	**Symbol**	**Value**	**Parameter**	**Symbol**	**Value**
Standard redox potential of ORR	$E^{0'}$	1.23 V vs RHE	Slope	$2.3RT/\alpha zF$	86 mV dec^{-1}
			Exchange current density	$j_{0\ \mathrm{exp}}$	7.0×10^{-9} A cm^{-2}

(7) ORR data 7

In a previous study[37], the ORR was studied using CcO-like Fe-N-C single atom nano enzymes with iron corrole (C@PVI-(TPC)Fe). The system used in the experiment is shown in the Figure S18(A) and it exhibited good selectivity for 4e- ORR and higher power density than Pt/C just as expected from CcO activity. To calculate the overpotential, the experimental data were extracted from the LSV plot in the Ref [37] - Fig. 4B. The original raw data shown in Figure S18 (B). In the reference, the coverage of active sites was not mentioned. Therefore, we estimated it as follows:

It was mentioned that the amount of Fe in C@PVI-(TPC) Fe-800 is 0.39 wt%. The catalyst loading in the reference was 1 mg cm^{-2}. Then, using the information of Fe molecular weight, 55.845 g mol^{-1}, σ was estimated

$$\sigma = \frac{(1 \times 10^{-3}\ \mathrm{g\ cm^{-2}})(0.0039)}{55.845\ \mathrm{g\ mol^{-1}}} = 6.9 \times 10^{-8}\ \mathrm{mol\ cm^{-2}}$$

The Tafel plot is shown in the Figure S18 (C) with $E^{0'}$ = 1.23 V vs RHE. The input and fitting parameters used in the tafel plot are shown in Table S18. Using this information, the overpotential for a specific Γ_e was estimated using the abovementioned method.

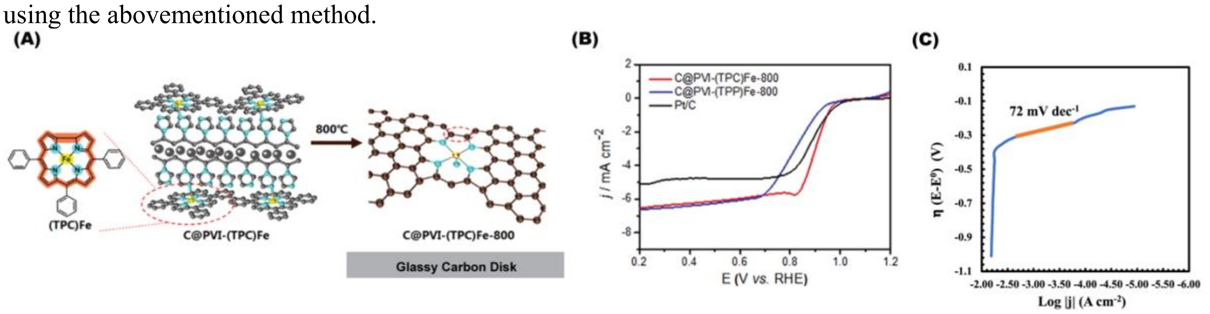

Figure S18. (A) Schematic illustration of the electrochemical system used in this study. (B) Original LSV from ref[37](Figure 4B); the C@PVI-(TPC)Fe-800 (the red line) data were used for calculation. (C) Replotted Tafel plot derived from (B) using $E^{0'}$ = 1.23 V. The orange line indicates the Tafel slope used for the overpotential calculation. Panels (A) and (B) was reprinted from ref [37]

Table S18. Input and fitted parameters used in the Tafel analysis for Figure S18

Input parameter from the reference[37]			Fitting parameter		
**Parameter**	**Symbol**	**Value**	**Parameter**	**Symbol**	**Value**
Standard redox potential of ORR	$E^{0'}$	1.23 V vs RHE	Slope	$2.3RT/\alpha zF$	72 mV dec^{-1}
			Exchange current density	$j_{0\ exp}$	1.2× 10^{-7} A cm^{-2}

S3. Estimation of the thermogenesis ratio at different electron transfer frequency (Γ_e)

The average value of the overpotential in each complex were calculated based on the calculation of all the data in Table S3. Then, total heat was obtained by took the sum of all the average overpotentials in each complex, as shown in eq. (5) below, where $\overline{\eta_1}$, $\overline{\eta_2}$, $\overline{\eta_3}$ and $\overline{\eta_4}$ refers to the average overpotential at a specific Γ_e in complex 1, 2, 3, and 4, respectively.

$$\text{Total Heat} = \sum(\overline{\eta_1} + \overline{\eta_2} + \overline{\eta_3} + \overline{\eta_4}) \quad (5)$$

The proportion of thermogenesis to all the energy gain can be calculated by dividing Total heat by 1.04 V, which corresponds to the total energy gain in the respiratory chain (shown in eq.(6))

$$\text{Thermogenesis Ratio} = \frac{\text{Total heat}}{1.04\ \text{V}} \times 100\% \quad (6)$$

By changing the Γ_e, thermogenesis and ATP proportions were obtained as shown in Fig. 2B in the main text

S4. ETF comparison between in-vivo and in-vitro

Table S19. Summary of in vivo oxygen consumption rate (OCR) measurements and corresponding electron transfer frequency (Γ_e) calculated from these data

No	Cell Name	OCR (mol s^{-1} mg-protein^{-1})	Γ_e (e$^-$ site^{-1} s^{-1})	Ref
1	Human skin	8.3E-12	0.20	48
2	Hepatocyte	1.3E-09	32.24	49
3	Brain	2.4E-11	0.57	50
4	Chick Cartilage	1.0E-11	0.25	
5	Skeletal Muscle	7.5E-13	0.02	51
6	Skeletal Muscle	3.7E-12	0.09	
7	Skeletal Muscle	3.9E-12	0.09	
8	Skeletal Muscle	6.4E-12	0.15	52
9	Human	1.1E-11	0.27	48
10	Human	7.5E-11	1.79	53
11	Leg muscle	6.3E-10	15.26	54

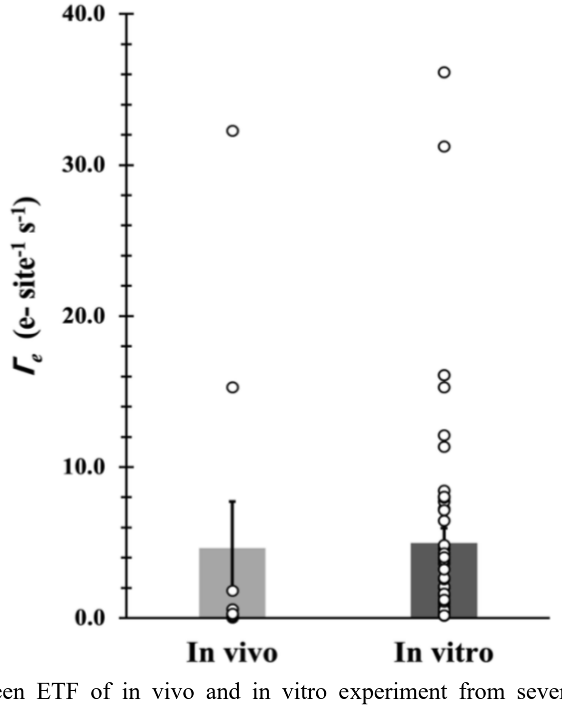

Figure S19 . Comparison between ETF of in vivo and in vitro experiment from several papers (See Supplementary Information). The bar graph shows the average of the overall data, the dot shows value of each data, and the line is the error bar between max and min value

S5. ETF of proton leak

Table S20. OCR of various UCP1-contained cells

No	Cell Name	OCR (mol/ s mg-protein)	Γ_e (e^- $site^{-1}$ s^{-1})	Ref
1	Beige adipocyte	1.67E-12	0.04	55
2		5.00E-12	0.12	
3	iBAT (Interscapular Brown Adipose Tissue)	3.67E-12	0.12	56
4		5.00E-12	0.09	
5		2.27E-10	5.46	57
6		4.33E-10	10.44	
7		4.17E-09	100.37	58
8	hBA (Clonal Human Brown preadipocytes)	3.00E-09	72.26	59
9	BAT	1.57E-10	3.79	60
10		2.50E-10	6.02	61
11		3.00E-10	7.23	62
12		3.33E-10	8.03	61
13		3.67E-10	8.83	63
14		4.17E-10	10.04	64
15		5.00E-10	12.04	65
16		5.00E-10	12.04	66
17		5.17E-10	12.45	60
18		6.67E-10	16.06	67
19		1.00E-09	24.09	68
20		1.67E-09	40.15	69
21		3.33E-09	80.29	70
22		6.33E-09	152.56	71

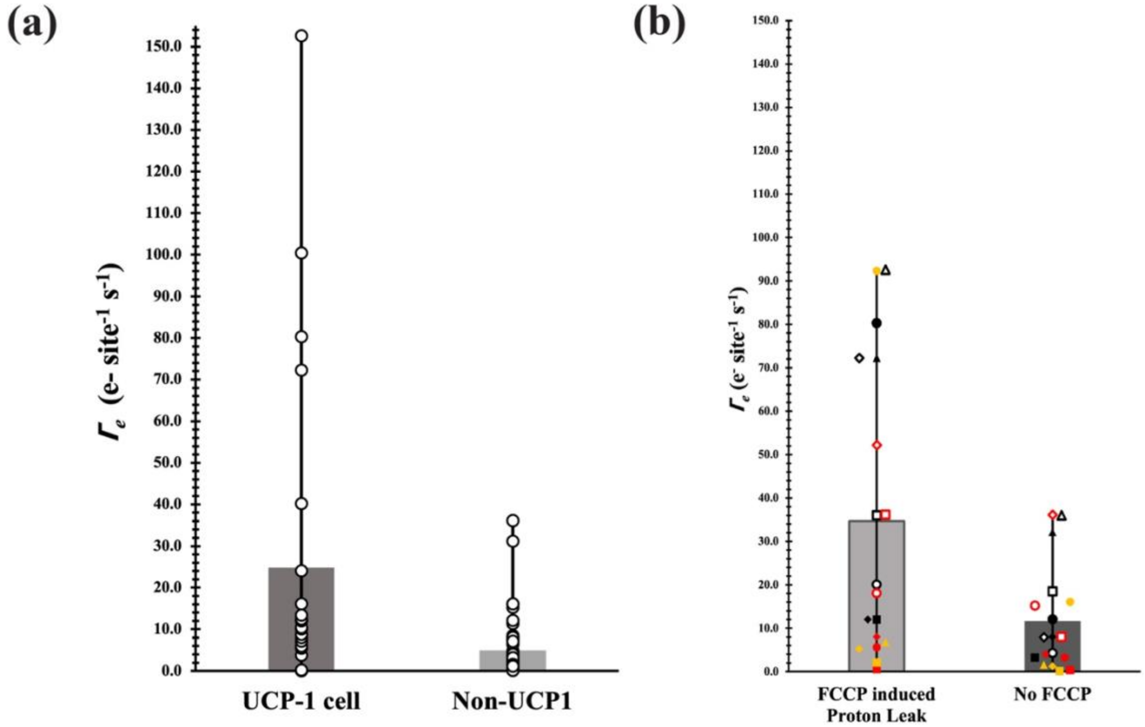

Figure S20. Comparison between ETF in the absence and presence of proton leak (FCCP and UCP1) (a) Comparison of ETF between UCP1 cell and non-UCP1 cell from several papers (see Table S20). The bar graph shows the average of the overall data, the dot shows value of each data, and the line is the error bar between max and min value. (b) Comparison of ETF in the presence and absence of FCCP from several papers (see Table S1 no 36-52). The bar graph shows the average of the overall data, the same symbol corresponds the data from the same reference and the line is the error bar between max and min value. The no FCCP data are taken from non UCP1 data that have FCCP experiment. Since the FCCP experiment data usually use high energy organ such as kidney, liver, etc, therefore the ETF is a little bit higher than non-UCP1 data.

S6. Sensitivity analysis

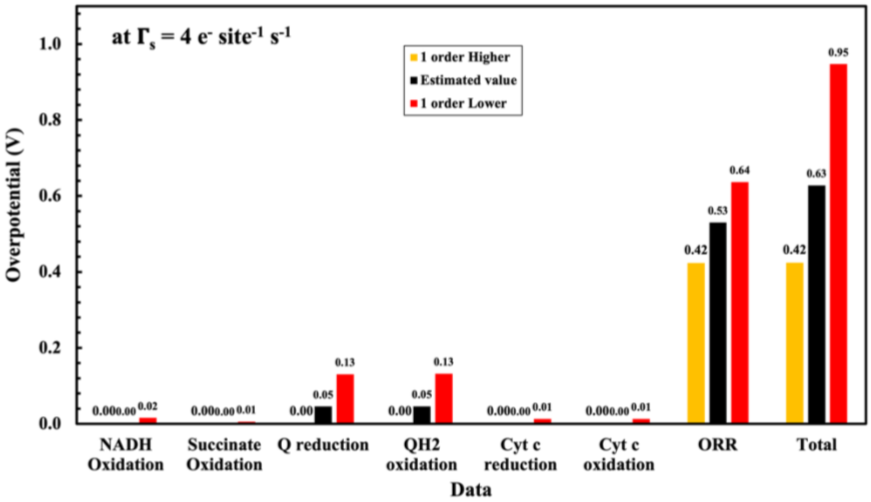

Figure S21. Sensitivity analysis of j_0 to the overpotential at 1 order higher (yellow) and 1 order lower (red) than the estimated j_0 (black).

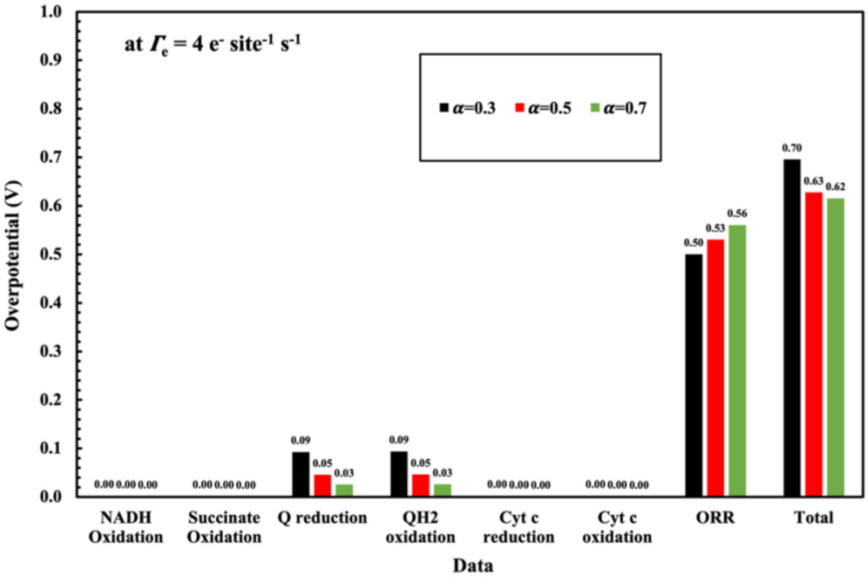

Figure S22. Sensitivity analysis of α to overpotential in each reaction and total overpotential. At $\alpha = 0.3$ (black), 0.5 (red), and 0.7 (green).

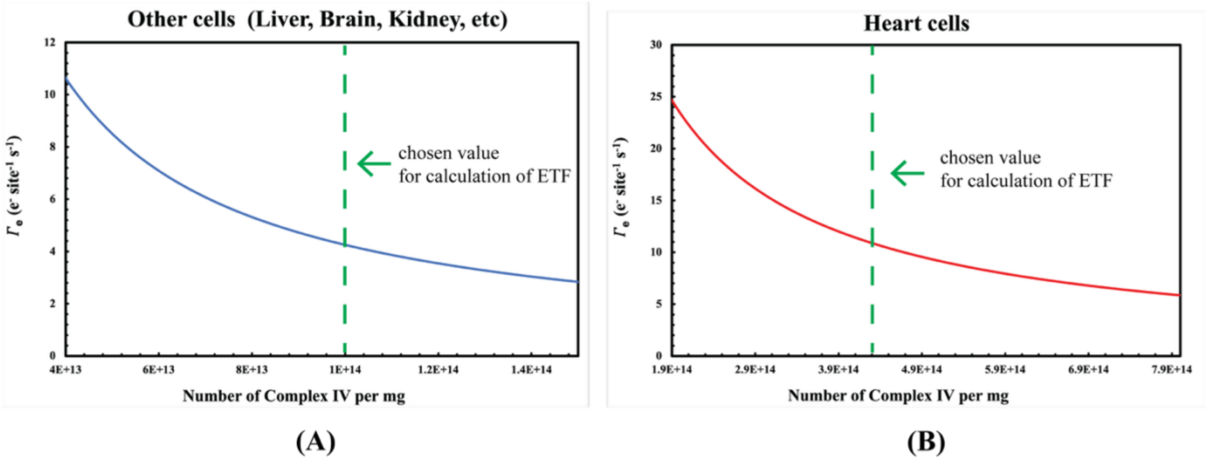

Figure S23. Sensitivity analysis of Number of Complex IV per mg to Electron transfer frequency (Γ_e). (a) Correlation between number of Complex IV per mg to Γ_e for cells other than heart cells. The range are taken from several papers (see Table S2) (b) Correlation between number of Complex IV per mg to Γ_e for heart cells. The range are taken from several papers (see Table S2), and green-dot-lines are the chosen value of number of complex IV per mg that we use in our calculation. The range of Number of Complex IV per mg in Heart cells is realatively higher than other cells.

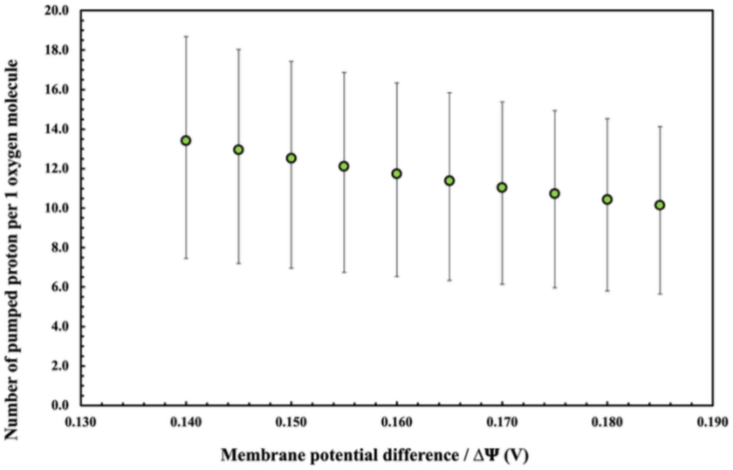

Figure S24. Impact of membrane potential to number of pumped proton. Correlation between different membrane potential to number of pumped proton per 1 oxygen molecule. $\Gamma_e = 4$ e^- per site per s was used in this calculation

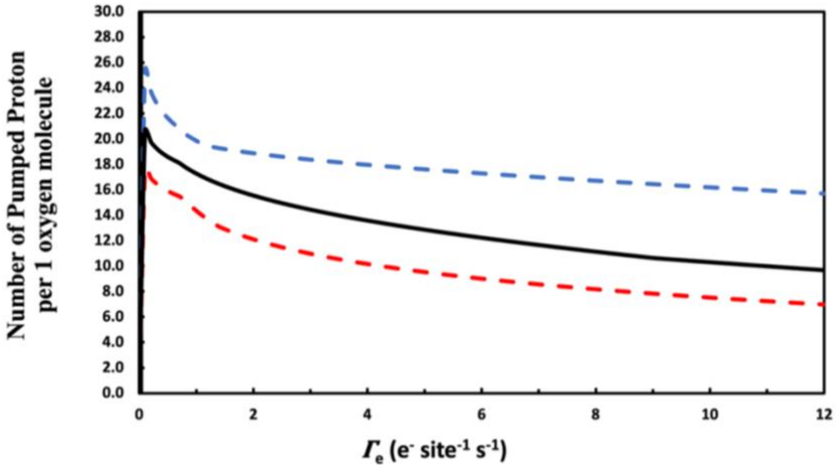

Figure S25. Model-predicted relationship between electron transfer frequency Γ_e and number of pumped proton per 1 oxygen molecule. The black line is the average value, the red line is the minimum range of error, and the blue line is the maximal range of error. $\Delta\Psi = 0.150$ was used in this calculation

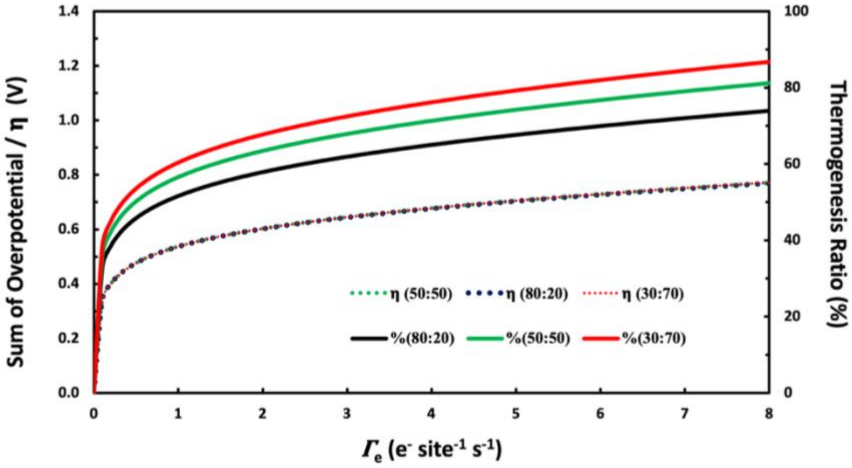

Figure S26. Sensitivity analysis showing the correlation between Γ_e , the overpotential (η), and the thermogenesis ratio at different Complex I:Complex II (CI:CII) ratios. Results are shown for CI:CII = 30:70 (red), 50:50 (green), and 80:20 (black). Dotted lines indicate the total overpotential, while solid lines represent the thermogenesis ratio under each conditions. The total overpotential does not change substantially, and the thermogenesis ratio varies by only ~10% as the succinate flux increase from 20% to 70%, indicating that this range of flux variation has only a minor impact on thermogenesis.

S7. Heat vs ETF

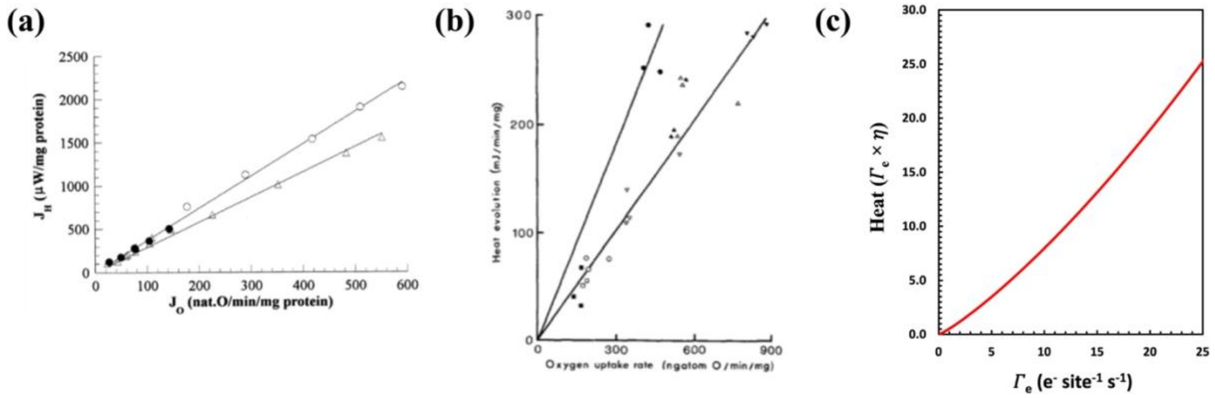

Figure S27. Comparison between experimentally observed and model-predicted relationships between mitochondrial respiration and heat production. (a) Correlation between heat generation rate (J_H) and oxygen uptake rate (J_O) for rat liver mitochondria under basal and uncoupled conditions. Adapted from ref [72] .(b) Relationship between oxygen uptake rate and heat evolution in E. coli membranes, showing enhanced heat production with increased respiratory activity. Reproduced from ref[73].(c) Theoretical prediction from the present overpotential-derived thermogenesis model. The normalized heat production increases nonlinearly with respiration rate at low activity and approaches linear dependence at high activity, reflecting the transition from kinetic-limited to diffusion-limited regimes of enzymatic electron transfer.

S8. Overpotential-driven thermogenesis based on Non-equilibrium thermodynamics

Here, overpotential-driven thermogenesis in mitochondria is discussed based on the non-equilibrium thermodynamics and entropy production concept founded by Ilya Prigogine[74]. The starting point of Prigogine theory is by expressing the changes in entropy as a sum of two parts:

$$\mathrm{d}S = \mathrm{d_e}S + \mathrm{d_i}S \tag{6-1}$$

where $\mathrm{d}S$ is the inside total variation of the entropy of a system, $\mathrm{d_e}S$ is the system's entropy change due to exchange of matter and energy with the exterior and $\mathrm{d_i}S$ is the entropy produced by the irreversible processes in the inside of the system. We may emphasize that one of the important objectives of non-equilibrium thermodynamics is to relate the entropy production $\mathrm{d_i}S$ to the driving force of various irreversible processes that occur in a system. The entropy production can thus serve as a basis for the systematic description of the irreversible processes occurring in a system.

Chemical reactions belong to this class of processes. Prigogine developed an explicit determination of the "uncompensated heat" (formulated by De Donder) by using $\mathrm{d_i}S75$[74]. For chemical processes in a closed system, $T\mathrm{d_i}S$ corresponds to Gibbs free energy terms at constant pressure and temperature.

$$T\mathrm{d_i}S = -\mathrm{d}G_{\mathrm{sys}} \tag{6-2}$$

where $\mathrm{d}G_{\mathrm{sys}}$ is the change of total Gibbs free energy of the reaction system. The driving force for the net reaction, $-\Delta G_{\mathrm{r}}$, can be obtained as the derivative the total Gibbs free energy of the reaction system with respect to the extent of reaction, ξ,

$$-\Delta G_{\mathrm{r}} \equiv -\frac{\mathrm{d}G_{\mathrm{sys}}}{\mathrm{d}\xi} \tag{6-3}$$

Then, one can relate the rate of entropy production to the driving force $-\Delta G_{\mathrm{r}}$ by combining equation (6-2), (6-3) and the rate of chemical reaction specifies $\mathrm{d}\xi/\mathrm{d}t$,

$$T\frac{\mathrm{d_i}S}{\mathrm{d}t} = -\frac{\mathrm{d}G_{\mathrm{sys}}}{\mathrm{d}t} = -\frac{\mathrm{d}G_{\mathrm{sys}}}{\mathrm{d}\xi}\frac{\mathrm{d}\xi}{\mathrm{d}t} = (-\Delta G_{\mathrm{r}})\,\frac{\mathrm{d}\xi}{\mathrm{d}t} \tag{6-4}$$

Also, the rate of entropy production corresponds to the generated Joule heat (per unit time) in electrical conduction system

$$T\frac{\mathrm{d_i}S}{\mathrm{d}t} = VI = \frac{\mathrm{d}Q'}{\mathrm{d}t} \tag{6-5}$$

where V is the potential difference across the entire conductor, I is the convention electric current, and $\mathrm{d}Q'$ is the Joule heat generated from the electric current. At steady-state, we know

$$\frac{\mathrm{d}S}{\mathrm{d}t} = \frac{\mathrm{d_e}S}{\mathrm{d}t} + \frac{\mathrm{d_i}S}{\mathrm{d}t} = 0 \tag{6-6}$$

Thus, borrowed from the concept of mixed-potential-driven catalysis we have developed,[75] the energy conversion in overpotential-driven thermogenesis in mitochondria can be described at steady-state as follows

$$T\frac{\mathrm{d_i}S}{\mathrm{d}t} = -\frac{\mathrm{d}G_{\mathrm{sys}}}{\mathrm{d}t} = (-\Delta G_{\mathrm{r}})\,\frac{\mathrm{d}\xi}{\mathrm{d}t} = \frac{(-\Delta G_{\mathrm{r}})}{F}\,Fv = (|\eta_1| + |\eta_2|\,)i_{\mathrm{mix}} = -T\frac{\mathrm{d_e}S}{\mathrm{d}t} = \frac{\mathrm{d}Q}{\mathrm{d}t} \tag{6-7}$$

where η_1, η_2 and i_{mix} are the overpotentials for half-reaction 1, 2 and current at mixed potential, $\mathrm{d}Q$ is the joule heat generated from reactions. Eq. (6-7) can be illustrated using the closed, isothermal, and isobaric biological enzymatic system depicted in Figure S26. The closed biological enzymatic system is at steady-state, while surroundings is enclosed by rigid adiabatic walls. We assume that surroundings achieve equilibrium throughout, meaning that temperature, pressure, and chemical potentials remain constant. This situation is often approximated experimentally.

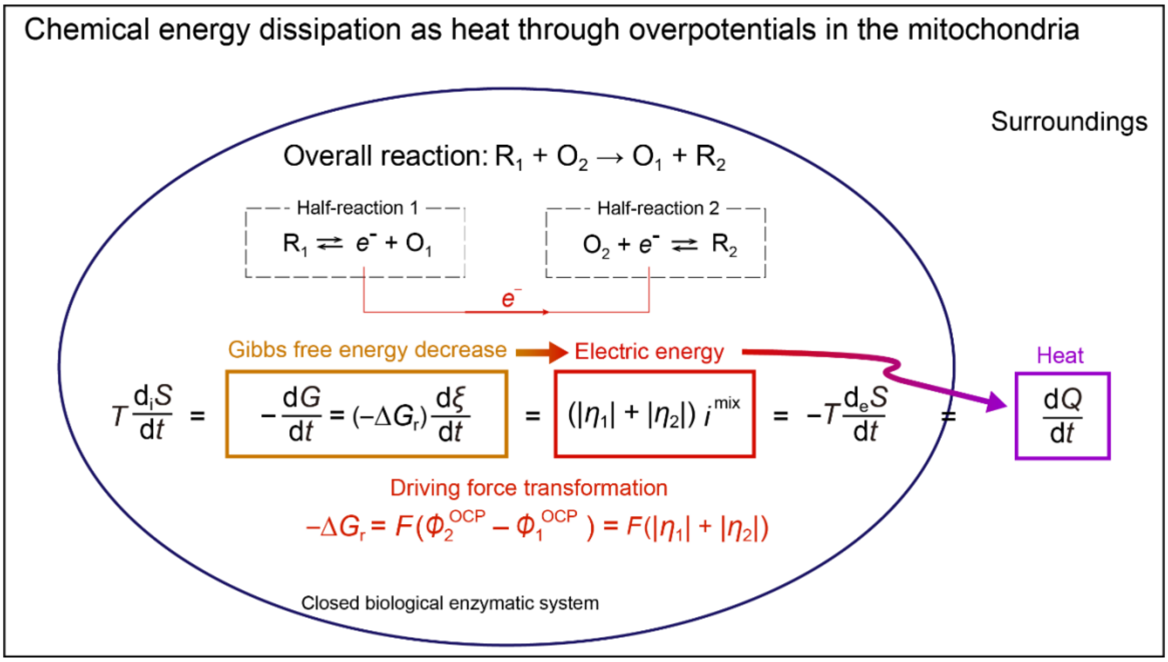

Figure S28. Steady-state biological enzymatic reactions occur in a closed, isothermal, and isobaric system, which is relatively small compared to its surroundings. Surroundings are enclosed by rigid adiabatic walls, completely isolated from the external world, a common experimental approximation. Adapted from Ref[75], Springer Nature, 2024, CC-BY 4.0.

One of the most important points here is that it provides a mechanism of transformation for the driving force where $-\Delta G_r$ is converted to overpotentials inside the reaction system. Then, one can conclude that, in the mitochondrial electron transport chain, it converts the Gibbs free energy decrease into internal electric energy through overpotentials, and eventually, this energy is dissipated as Joule heat.

References:

1 B. A. Wagner, S. Venkataraman and G. R. Buettner, *Free Radic. Biol. Med.*, 2011, **51**, 700–712.

2 E. Bianconi, A. Piovesan, F. Facchin, A. Beraudi, R. Casadei, F. Frabetti, L. Vitale, M. C. Pelleri, S. Tassani, F. Piva, S. Perez-Amodio, P. Strippoli and S. Canaider, *Ann. Hum. Biol.*, 2013, **40**, 463–471.

3 R. Z. Zhao, X. B. Wang, S. Jiang, N. Y. Ru, B. Jiao, Y. Y. Wang and Z. Bin Yu, *Pflugers Arch.*, 2020, **472**, 1619–1630.

4 D. Roussel, N. Roussel, Y. Voituron and B. Rey, *Mitochondrion*, 2024, **78**, 101909.

5 P. Rybkowska, K. Radoszkiewicz, M. Kawalec, D. Dymkowska, B. Zabłocka, K. Zabłocki and A. Sarnowska, *Cells*, 2023, **12**, 178.

6 A. McCrimmon, M. Domondon, R. F. Sultanova, D. V Ilatovskaya and K. Stadler, *American Journal of Physiology-Renal Physiology*, 2020, **318**, F1237–F1245.

7 H. Fuchs, A. Malecka, A. Budzinska, W. Jarmuszkiewicz, L. Ciszewska, A. M. Staszak, J. Kijowska-Oberc and E. Ratajczak, *BMC Plant Biol.*, 2023, **23**, 496.

8 P. Brzęk, D. Roussel and M. Konarzewski, *Proceedings of the Royal Society B: Biological Sciences*, 2022, **289**, 20220719.

9 J. M. Son, E. H. Sarsour, A. Kakkerla Balaraju, J. Fussell, A. L. Kalen, B. A. Wagner, G. R. Buettner and P. C. Goswami, *Aging Cell*, 2017, **16**, 1136–1145.

10 J. Vine, J. H. Lee, L. Balaji, A. V Grossestreuer, A. Morton, N. Peradze, N. Antony, N. Berlin, M. S. Kravitz, S. B. Leland, K. Berg, A. Moskowitz, M. W. Donnino and X. Liu, *Intensive Care Med. Exp.*, 2024, **12**, 97.

11 G. W. Rogers, M. D. Brand, S. Petrosyan, D. Ashok, A. A. Elorza, D. A. Ferrick and A. N. Murphy, *PLoS One*, 2011, **6**, e21746-.

12 L. G. Corales, H. Inada, Y. Owada and N. Osumi, *Genes to Cells*, 2024, **29**, 757–768.

13 R. A. Capaldi, D. G. Halphen, Y.-Z. Zhang and W. Yanamura, *J. Bioenerg. Biomembr.*, 1988, **20**, 291–311.

14 T. E. King, K. S. Nickel and D. R. Jensen, *J. Biol. Chem.*, 1964, **239**, 1989–1994.

15 P. V Blair, T. Oda, D. E. Green, H. FERNANDEZ-MORAN Biochemistry Plaut, J. Biol Chem and M. General Hospital, *Biochemistry*, 1963, **2**, 756–763.

16 A. D. Vinogradov and T. E. King, *Methods Enzymol.*, 1979, **55**, 118–127.

17 G. F. Azzone, R. Colonna and B. Ziche, *Methods Enzymol.*, 1979, **55**, 46–50.

18 R. S. Balaban, V. K. Mootha and A. Arai, *Anal. Biochem.*, 1996, **237**, 274–278.

19 G. Benard, B. Faustin, E. Passerieux, A. Galinier, C. Rocher, N. Bellance, J.-P. Delage, L. Casteilla, T. Letellier, R. Rossignol and R. Ros, *Am J Physiol Cell Physiol*, 2006, **291**, 1172–1182.

20 A. Tanaka, T. Morimoto, S. Wakashiro, I. Ikai, K. Ozawa and Y. Orii, *Life Sci.*, 1987, **41**, 741–748.

21 K. Schwerzmann, L. M. Cruz-Orive, R. Eggman, A. S~inger and E. R. Weibel, *J. Cell Biol.*, 1986, **102**, 97–103.

22 C. R. Hackenbrock, B. Chazotte and S. Shaila Gupte, *J. Bioenerg. Biomembr.*, 1986, **18**, 331–368.

23 P. Padma and O. H. Setty, *J. Clin. Biochem. Nutr*, 1998, **24**, 35–43.

24 C. D. Barker, T. Reda and J. Hirst, *Biochemistry*, 2007, **46**, 3454–3464.

25 A. Sucheta, B. A. C. Ackrell, B. Cochran and F. A. Armstrong, *Ltters to Nature*, 1992, **356**, 361–362.

26 O. Kolaj-Robin, S. R. O'Kane, W. Nitschke, C. Léger, F. Baymann and T. Soulimane, *Biochim. Biophys. Acta Bioenerg.*, 2011, **1807**, 68–79.

27 S. Srinivas, K. Ashokkumar, K. Sriraghavan and A. Senthil Kumar, *Sci. Rep.*, 2021, **11**, 13905.
28 D. Arthisree, K. S. S. Devi, S. L. Devi, K. Meera, G. M. Joshi and A. Senthil Kumar, *Colloids Surf. A Physicochem. Eng. Asp.*, 2016, **504**, 53–61.
29 M. Gandhi, D. Rajagopal and A. Senthil Kumar, *Electrochim. Acta*, 2021, **368**, 137596.
30 S. Ray, D. Yadav, S. S. Garje and S. Mazumdar, *Journal of Chemical Sciences*, 2021, **133**, 94.
31 E. Lebègue, H. Smida, T. Flinois, V. Vié, C. Lagrost and F. Barrière, *Journal of Electroanalytical Chemistry*, 2018, **808**, 286–292.
32 S. Chatterjee, K. Sengupta, S. Hematian, K. D. Karlin and A. Dey, *J. Am. Chem. Soc.*, 2015, **137**, 12897–12905.
33 S. Mukherjee, A. Mukherjee, A. Bhagi-Damodaran, M. Mukherjee, Y. Lu and A. Dey, *Nat. Commun.,*2015, **6**, 8467.
34 S. Bhunia, A. Rana, P. Roy, D. J. Martin, M. L. Pegis, B. Roy and A. Dey, *J. Am. Chem. Soc.*, 2018, **140**, 9444–9457.
35 C. Du, Y. Gao, H. Chen, P. Li, S. Zhu, J. Wang, Q. He and W. Chen, *J. Mater. Chem. A Mater.*, 2020, **8**, 16994–17001.
36 H. Zhang, L. Huang, J. Chen, L. Liu, X. Zhu, W. Wu and S. Dong, *Nano Energy*, 2021, **83**, 105798.
37 C. Xu, Y.-P. Zhang, T.-L. Zheng, Z.-Q. Wang, Y.-M. Zhao, P.-P. Guo, C. Lu, K.-Z. Yang, P.-J. Wei, Q.-G. He, X.-Q. Gong and J.-G. Liu, *ACS Appl. Mater. Interfaces*, 2023, **15**, 32341–32351.
38 J. H. Brown, *J. Chem. Educ.*, 2015, **92**, 1490–1496.
39 A. Rohatgi, *WebPlotDigitizer*, https://automeris.io/WebPlotDigitizer, version 4.5
40 Y. Zhao, Y. Yang and J. Loscalzo, in *Methods in Enzymology*, Academic Press Inc., 2014, vol. 542, 349–367.
41 L. Tretter, A. Patocs and C. Chinopoulos, *Biochim. Biophys. Acta Bioenerg.*, 2016, **1857**, 1086–1101.
42 G. Lenaz and M. L. Genova, *Biochim. Biophys. Acta Bioenerg.*, 2009, **1787**, 563–573.
43 K. Aoki, *Review of Polarography*, 2003, **49**, 103–105.
44 S. Ray, D. Yadav, S. S. Garje and S. Mazumdar, *J.Chem.Schi.*
45 H. Jay Forman and A. AZZItI, *The FASEB Journal*, 1997, **11**, 374–375.
46 E. Gnaiger, in *Hypoxia*, eds. R. C. Roach, P. D. Wagner and P. H. Hackett, Springer US, Boston, MA, 2003, 39–55.
47 C. Wei, R. R. Rao, J. Peng, B. Huang, I. E. L. Stephens, M. Risch, Z. J. Xu and Y. Shao-Horn, *Advanced Materials*, 2019, **31**, 1806296.
48 M. A. Wefers Bettink, J. Zwaag, B. Schockaert, P. Pickkers, M. Kox and E. G. Mik, *Sci. Rep.*, 2025, **15**, 25815.
49 E. G. Mik, T. Johannes, C. J. Zuurbier, A. Heinen, J. H. P. M. Houben-Weerts, G. M. Balestra, J. Stap, J. F. Beek and C. Ince, *Biophys. J.*, 2008, **95**, 3977–3990.
50 R. Milo, P. Jorgensen, U. Moran, G. Weber and M. Springer, *Nucleic Acids Res.*, 2010, **38**, D750–D753.
51 K. Gurley, Y. Shang and G. Yu, *J. Biomed. Opt.*, 2012, **17**, 075010.
52 W. H. Nugent, B. K. Song, R. N. Pittman and A. S. Golub, *Microvasc. Res.*, 2016, **105**, 15–22.
53 G. Villani and G. Attardi, *Proceedings of the National Academy of Sciences*, 1997, **94**, 1166–1171.

54 J. R. Gifford, R. S. Garten, A. D. Nelson, J. D. Trinity, G. Layec, M. A. H. Witman, J. C. Weavil, T. Mangum, C. Hart, C. Etheredge, J. Jessop, A. Bledsoe, D. E. Morgan, D. W. Wray, M. J. Rossman and R. S. Richardson, *J. Physiol.*, 2016, **594**, 1741–1751.

55 E. Nyman, S. Bartesaghi, R. Melin Rydfalk, S. Eng, C. Pollard, P. Gennemark, X.-R. Peng and G. Cedersund, *NPJ Syst. Biol. Appl.*, 2017, **3**, 29.

56 M. Calderon-Dominguez, M. Alcalá, D. Sebastián, A. Zorzano, M. Viana, D. Serra and L. Herrero, *Advanced Science*, 2017, **4**, 1600274.

57 B. E. Levin, M. B. Finnegan, E. Marquet and A. C. Sullivan, *American Journal of Physiology-Endocrinology and Metabolism*, 1984, **247**, E94–E100.

58 H. F. Raposo, P. Forsythe, B. Chausse, J. Z. Castelli, P. M. Moraes-Vieira, V. S. Nunes and H. C. F. Oliveira, *Metabolism*, 2021, **114**, 154429.

59 C. Cero, W. Shu, A. L. Reese, D. Douglas, M. Maddox, A. P. Singh, S. L. Ali, A. R. Zhu, J. M. Katz, A. E. Pierce, K. T. Long, N. Nilubol, R. H. Cypess, J. L. Jacobs, F. Tian and A. M. Cypess, *Endocrinology*, 2023, **164**, bqad161.

60 S. Schweizer, J. Oeckl, M. Klingenspor and T. Fromme, *Life Sci. Alliance*, 2018, **1**, e201800136.

61 A. Song, W. Dai, M. J. Jang, L. Medrano, Z. Li, H. Zhao, M. Shao, J. Tan, A. Li, T. Ning, M. M. Miller, B. Armstrong, J. M. Huss, Y. Zhu, Y. Liu, V. Gradinaru, X. Wu, L. Jiang, P. E. Scherer and Q. A. Wang, *Journal of Clinical Investigation*, 2020, **130**, 247–257.

62 A. Bugge, L. Dib and S. Collins, *Methods Enzymol.*, 2014, **538**, 233–247.

63 L. Li, A. M. Gunewardena, T. Nyima and B. J. Feldman, *STAR Protoc.*, 2023, **4**, 102607.

64 D. P. Blondin, S. Nielsen, E. N. Kuipers, M. C. Severinsen, V. H. Jensen, S. Miard, N. Z. Jespersen, S. Kooijman, M. R. Boon, M. Fortin, S. Phoenix, F. Frisch, B. Guérin, É. E. Turcotte, F. Haman, D. Richard, F. Picard, P. C. N. Rensen, C. Scheele and A. C. Carpentier, *Cell Metab.*, 2020, **32**, 287-300.e7.

65 M. Bauzá-Thorbrügge, M. Vujičić, B. Chanclón, V. Palsdottir, N. J. Pillon, A. Benrick and I. Wernstedt Asterholm, *Metabolism*, 2024, **151**, 155716.

66 S. Rajan, K. Shankar, M. Beg, S. Varshney, A. Gupta, A. Srivastava, D. Kumar, R. K. Mishra, Z. Hussain, J. R. Gayen and A. N. Gaikwad, *Journal of Endocrinology*, 2016, **230**, 275–290.

67 M. Abelenda, C. Castro, C. Venero and M. Puerta, *Can. J. Physiol. Pharmacol.*, 1994, **72**, 1226–1230.

68 J. L. Barclay, H. Agada, C. Jang, M. Ward, N. Wetzig and K. K. Y. Ho, *Journal of Endocrinology*, 2015, **224**, 139–147.

69 A. Park, K. Kim, I. Park, S. H. Lee, K.-Y. Park, M. Jung, X. Li, M. B. Sleiman, S. J. Lee, D.-S. Kim, J. Kim, D.-S. Lim, E.-J. Woo, E. W. Lee, B. S. Han, K.-J. Oh, S. C. Lee, J. Auwerx, J. Y. Mun, H.-W. Rhee, W. K. Kim, K.-H. Bae and J. M. Suh, *Nat. Commun.*, 2023, **14**, 3746.

70 N. M. Held, E. N. Kuipers, M. van Weeghel, J. B. van Klinken, S. W. Denis, M. Lombès, R. J. Wanders, F. M. Vaz, P. C. N. Rensen, A. J. Verhoeven, M. R. Boon and R. H. Houtkooper, *Sci. Rep.*, 2018, **8**, 9562.

71 Y. Qiu, L. Sun, X. Hu, X. Zhao, H. Shi, Z. Liu and X. Yin, *Diabetol. Metab. Syndr.*, 2020, **12**, 91.

72 L. Dejean, B. Beauvoit, O. Bunoust, C. Fleury, B. Guérin and M. Rigoulet, *Biochimica et Biophysica Acta (BBA) - Bioenergetics*, 2001, **1503**, 329–340.

73 R. K. Poole and B. A. Haddock, *FEBS Lett.*, 1975, **58(1)**, 249.

74 D. K. . Kondepudi and I. . Prigogine, *Modern thermodynamics : from heat engines to dissipative structures*, John Wiley & Sons, 1999.

75 M. Yan, N. A. P. Namari, J. Nakamura and K. Takeyasu, *Commun. Chem.*, 2024, **7**, 69.
